# A epidemiological trend of chronic kidney disease due to hypertension among adolescents and young adults: global burden and future 2035 projections

**DOI:** 10.3389/fpubh.2025.1618416

**Published:** 2025-08-04

**Authors:** Tao Wang, Rui Pan, Cong Li, Ying Qin, Chao Song

**Affiliations:** ^1^Department of Critical Care Medicine, Guiqian International General Hospital, Guiyang, Guizhou, China; ^2^Department of Anesthesia, Meitan County Maternal and Child Health Hospital, Zunyi, Guizhou, China

**Keywords:** chronic kidney disease, hypertension, adolescents, Global Burden of Disease 2021, health policy, epidemiology

## Abstract

**Background:**

The study aimed to describe the epidemiological trend of chronic kidney disease (CKD) due to hypertension among adolescents and young adults during 1990–2021 worldwide. Additionally, the study seeks to provide comprehensive estimate of the associated risk factors for mortality and to project the burden of disease over the next decade.

**Methods:**

We utilized the Global Burden of Disease (GBD) to assess the changing trends of the standardized incidence rate (ASIR), death (ASDR), and disability-adjusted life years (DALYs) ASR by calculating the estimated average percentage change (EAPC). Meanwhile, to assesses proportional death of CKD due to hypertension attributable to associated risk factors. The Bayesian Age-Time-Quest Model (BAPC) to predict the ASIR, ASDR and DALY ASR for young people aged 15 to 39 by 2035.

**Results:**

In 2021, there were 36,754 incident cases of CKD due to hypertension among adolescents and young adults worldwide. The corresponding ASIR of CKD due to hypertension was 0.91 per 100,000 population (95% uncertainty interval [UI], 0.67–1.18), and the EAPC was 0.99(95% Confidence interval (CI), 0.95–1.03) from 1990 to 2021. The DALYs case of CKD due to hypertension increased from 825,628.28(95%UI, 646,823.70–1,054,246.62) to 1,180,452.47(95%UI, 889,946.51–1,523,802.55), and the corresponding ASR increased from 37.67 per 100,000 population (95%UI, 29.51–48.10) to 39.68 per 100,000 population (95%UI, 29.92–51.22), the EAPC was −0.01(95% CI, −0.10 to 0.08). Among the 5 SDI regions, the middle SDI region had the highest ASIR of CKD due to hypertension in 2021. Regionally, High-income North America had the largest increase in ASDR (EAPC, 3.92; 95% CI, 3.58 to 4.26). Among 204 countries, Nicaragua had the highest national ASIR of CKD due to hypertension in 2021 (4.47 per 100,000 population; 95% UI, 2.48–7.31), Finland had the lowest ASDR (0.00 per 100,000 population; 95% UI, 0.00–0.00). Globally, dietary risks kidney dysfunction, high systolic blood pressure, were key risk factors for CKD due to hypertension-associated mortality in 2021. By 2035 years, ASDR and DALYs related to CKD due to hypertension will decline worldwide, ASIR are projected to continue rising among adolescents and young adults.

**Conclusion:**

CKD resulting from hypertension globally poses a significant challenge for healthcare systems. Therefore, a comprehensive understanding of the epidemiological characteristics of CKD associated with hypertension will be crucial for developing more effective prevention and control strategies.

## Introduction

As the global population continues to rise, chronic kidney disease (CKD) poses a significant challenge to public health, the number of CKD cases worldwide approached 700 million by 2017 (1). In recent years, due to the gradual rejuvenation of more and more diseases such as diabetes and cardiovascular disease (2, 3). Consequently, CKD has emerged as a disease that particularly affects adolescents and young adults. Key contributing factors include poor lifestyle choices, obesity, and unhealthy dieting practices (4, 5). According to the 2019 Global Burden of Disease, Injuries, and Risk Factor Study (GBD), the incidence, mortality, and disability-adjusted life years (DALYs) rates for CKD among adolescents and young adults from 1990 to 2019 were reported as 32.21 per 100,000 population, 2.86 per 100,000 population, and 236.85 per 100,000 population (6), respectively, indicating a continuing upward trend. Therefore, a timely understanding of the global burden of kidney disease in young adults is essential for policymakers to effectively implement primary prevention and control strategies.

Hypertension is recognized as the primary risk factor for CKD (7). Reports indicate that hypertension affects up to 90% of individuals with CKD and contributes to the progression of the disease (8, 9). The damage to the glomerular arteries manifests as arteriosclerosis, resulting in lumen narrowing and decreased blood flow, which is a principal cause of renal abnormalities associated with hypertension (7). Increasingly, younger populations are being affected by hypertension, leading to kidney damage and the development of CKD. While some experts advocate for maintaining blood pressure within the range of 130–140 mmHg, a consensus has yet to be achieved, complicating the management of CKD (10). Furthermore, the distribution of hypertensive CKD varies globally, with the mortality rate in low-income countries being particularly high (11). Thus, the formulation and implementation of effective, targeted prevention strategies for hypertension-related CKD is a critical issue that the world faces.

According to the GBD report, the age-standardized incidence rate (ASIR) of global chronic kidney disease due to hypertension increased from 15.97 to 19.45 per 100,000 population, while the age-standardized death rate (ASDR) rose from 5.1 to 5.88 per 100,000 population from 1990 to 2019 (12). However, the specific risk factors and global burden of CKD attributable to hypertension in young adults have yet to be clearly identified. These research gaps may impede policymakers from formulating effective strategies at both regional and national levels to address CKD associated with hypertension in this demographic. In this study, utilizing the Global Burden of Disease (GBD) 2021 database, we determined the ASIR, ASDR and disability-adjusted life years (DALYs) ASR of hypertension-related CKD among adolescents and young adults aged 15 to 39 years across 204 countries at global, regional, and national levels from 1990 to 2021. We analyzed epidemiological trends and identified attributable risk factors. Additionally, we projected trends extending to 2035, with data categorized by age, gender, region, country, and Sociodemographic Index (SDI). We expect this study to the prevention and treatment of hypertension-related CKD in adolescents and young people, ultimately reducing the risk of mortality associated with the disease.

## Methods

### Data collection

Data for this research were sourced from the 2021 Global Burden of Disease (GBD) dataset, offering extensive details about the worldwide and regional impact of 371 diseases, injuries, and 88 risk factors across 204 nations and territories spanning from 1990 to 2021 (13, 14). In this investigation, we collected data on the incidence, mortality, and DALYs of hypertension-related CKD among adolescents and young adults aged 15 to 39, along with their respective ASR at global, regional, and national levels. We calculated the average estimated annual percentage change (EAPC) using linear regression analysis. The data were retrieved and downloaded from the Global Health Data Exchange (GHDx) platform.^1^ Additionally, Sociodemographic Index (SDI) data were collected to evaluate how socioeconomic variables influence disease burden. We focused on CKD due to hypertension with an age range of 15–39 years (i.e., 15–19 years as youths, 20–39 as young adults) as per earlier publications using GBD data (3, 6).

### Definition

The SDI serves as a measure of a nation’s socioeconomic standing. It is derived by calculating the geometric mean of several indicators, including the average education level of individuals aged 15 and older, the total fertility rate for those below 25 years, and the distribution of per capita income, SDI (high, high-middle, middle, low-middle, and low SDI categories) (15).

### Statistical

All data presented in the figures and tables throughout this manuscript were sourced from the GBD 2021 database, ensuring authenticity and reliability. Following extraction, the data were re-analyzed and visualized through bar charts, line graphs, and map utilized software package (version 4.2.3) and JD_GBDR (V2.24, Jingding Medical Technology Co., Ltd.) categorized by age group, sex, and geographic region to characterize the epidemiological trend of hypertension-related CKD among adolescents and young adults. We utilized R software package (version 4.2.3) and JD_GBDR (V2.24, Jingding Medical Technology Co., Ltd.) to generate maps. These maps utilized the `rnaturalearthdata` packages to display the distribution of the disease burden. All estimated values for the age-standardized rate, number of cases, and fluctuations in case numbers are presented with a 95% uncertainty interval (UI), which is defined as the range between the 2.5th and 97.5th percentiles among all 1,000 simulations.

The age-standardized rates can exclude the effects of imbalances in population size and age distribution, $\mathrm{ASR}=\frac{\sum_{i=1}^{A}\;a_{i}w_{i}}{\sum_{i=1}^{A}\;a_{i}}\times 100000$ . In addition, the spatial and temporal trends in the disease burden of acute pancreatitis, CKD due to hypertension were captured by using the estimated annual percentage change (EAPC) corresponding to the age-standardized incidence and death rate per 100,000 population (ASIR/ASDR) and the age-standardized DALY rate. Y = *α* + *β* X where Y is the lg (age-standardized rate) and X is the calendar year. The EAPC value was then calculated by the formula EAPC = 100 * (exp(β)-1). the EAPC is presented along with a 95% confidence interval (CI) to illustrate the magnitude and direction of temporal trends. Its 95% CI are greater than zero, the corresponding age-specification rate tends to increase, and vice versa (16, 17). Gaussian curves were used to analyze associations between EAPC and rates and the Human Development Index of CKD due to hypertension in adolescents and young adults. To ensure the accuracy and robustness of the predictions, we utilize data on CKD attributable to hypertension in adolescents and young adults from 1990 to 2021.

### Projective analyze

To project future disease burden, we implemented a Bayesian Age-Period-Cohort (BAPC) model (18), decomposing temporal trends into three dimensions: age (biological risk progression), period (time-specific external factors), and cohort (birth year-linked exposures). The number of cases is modeled through the use of the age-period-cohort (APC) framework, with age group A and period P:

$\log(Y_{\mathrm{ap}})=\mu +\alpha_{a}+\beta_{p}+\gamma_{c}$

In this model, μ denotes the intercept term, while *α_a_*, *β_p_* and *γ_c_* represent the age, period, and cohort effects in the log scale, respectively. Bayesian estimation is performed using the Integrated Nested Laplace Approximation (INLA), which assumes that the second-order differences of age, period, and cohort effects follow independent zero-mean normal distributions, thereby ensuring parameter smoothness. The effect of period *t*, *βp+tβp+t,* is derived based on historical trends:

βp+t∣βp,βp−1∼N((1+t)βp−tβp−1,kβ−1i=1∑ti2)

Consequently, the introduction of a random effect terms, δap + t ∼ N(0,kδ − 1)*δap*+*t* ∼ N(0,*kδ*−1) was implemented to calibrate the model residuals.

Prior distributions for model parameters were assigned using the Integrated Nested Laplace Approximation (INLA) algorithm, balancing historical data robustness with predictive flexibility. Model validation included 10-fold cross-validation on 1990–2010 data, with prediction accuracy assessed via mean absolute percentage error (MAPE). The BAPC demonstrated superior performance over non-Bayesian alternatives, evidenced by a 15.7% reduction in Deviance Information Criterion (DIC). Sensitivity analyses confirmed stability across prior distribution assumptions (coefficient variation <5%). All analyses were conducted using the “BAPC” R package (v1.0.2).

## Result

### CKD due to hypertension in adolescents and young adults: global trends

#### Incidence

**Figure 1:**
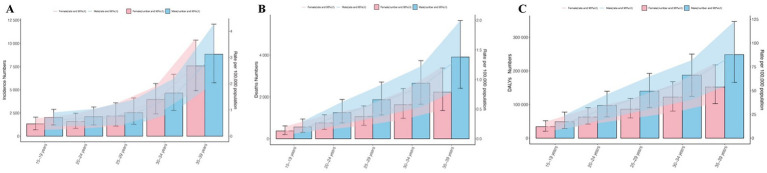
Trends in CKD due to hypertension incidence, deaths, and disability-adjusted life-years (DALYs) among adolescents and young adults in 2021. **(A)** Trends in incident cases and incidence rate. **(B)** Trends in death cases and death rate. **(C)** Trends in DALYs cases and DALYs rate.

From 1990 to 2021, the global incidence of CKD attributed to hypertension in adolescents and young adults rose significantly, increasing from 19,849 to 36,754 cases. The ASIR rose correspondingly from 0.91 per 100,000 (95% UI, 0.67–1.18) in 1990 to 1.24 per 100,000 (95% UI, 0.96–1.56) in 2021. The EAPC was 0.99 (95% CI, 0.95 to1.03). Within this demographic, the number of affected males surged from 10,596 to 20,145, while the number of affected females increased from 9,253 to 16,609. The incidence of CKD due to hypertension in adolescents and young adults is consistently higher in males than in females, and this incidence increases with age. For instance, the incidence of CKD due to hypertension was highest among adults aged 34 to 39 years in both 1990 and 2021, at 2.31 per 100,000 and 2.93 per 100,000, respectively. From 1990 to 2021, the ASIR increased by 24.78% for females and 27.82% for males (as shown in Table 1, Figure 1A, Supplementary Figure 1A).

**Table 1:** Incidence cases and ASIR of CKD due to hypertension in 1990 and 2021, and temporal trends.

	1990	ASIR (per 100,000 population)	2021	ASIR (per 100,000 population)	1990–2021
	Incidence cases (95% UI)		Incidence cases (95% UI)		EAPCs of ASR (95% CI)
Global	19849.19 (14719.58–25805.45)	0.91 (0.67–1.18)	36753.78 (28504.14–46380.72)	1.24 (0.96–1.56)	0.99 (0.95–1.03)
Sex					
Male	10596.40 (7958.88–13,722.46)	0.96 (0.72–1.24)	20144.98 (15,720.54–25,241.17)	1.33 (1.04–1.67)	1.11 (1.06–1.15)
Female	9252.78 (6,752.69–12,110.81)	0.85 (0.62–1.12)	16608.80 (12,802.02–21,074.24)	1.13 (0.87–1.44)	0.85 (0.81–0.89)
Socio-demographic index					
Low SDI	1436.52 (1075.10–1880.27)	0.78 (0.58–1.02)	4199.87 (3174.57–5304.83)	0.94 (0.71–1.18)	0.60 (0.50–0.70)
Low-middle SDI	4677.60 (3550.08–6025.45)	1.03 (0.78–1.33)	10514.98 (8163.62–13085.36)	1.31 (1.02–1.63)	0.72 (0.63–0.81)
Middle SDI	7479.31 (5628.56–9731.26)	0.99 (0.75–1.29)	13788.87 (10675.16–17317.09)	1.49 (1.15–1.87)	1.31 (1.28–1.34)
High-middle SDI	3919.07 (2875.79–5157.43)	0.87 (0.64–1.14)	5267.04 (3830.45–6943.11)	1.20 (0.87–1.58)	1.04 (0.96–1.12)
High SDI	2317.34 (1563.38–3314.48)	0.67 (0.45–0.96)	2948.83 (1961.22–4050.57)	0.83 (0.56–1.15)	0.64 (0.56–0.71)
Region					
Andean Latin America	114.14 (69.02–175.44)	0.74 (0.45–1.13)	330.92 (216.65–482.51)	1.22 (0.80–1.78)	1.76 (1.72–1.81)
Australasia	32.48 (17.40–53.21)	0.40 (0.21–0.65)	60.12 (33.83–93.93)	0.57 (0.32–0.90)	1.07 (0.92–1.22)
Caribbean	174.33 (118.57–242.91)	1.17 (0.80–1.63)	353.92 (250.27–478.25)	1.94 (1.37–2.63)	1.63 (1.48–1.78)
Central Asia	440.95 (298.28–638.23)	1.55 (1.05–2.24)	813.03 (556.80–1178.31)	2.17 (1.49–3.15)	1.03 (0.88–1.18)
Central Europe	417.82 (278.74–576.59)	0.89 (0.59–1.23)	416.43 (286.90–570.15)	1.19 (0.82–1.63)	1.04 (0.98–1.09)
Central Latin America	934.67 (661.07–1241.33)	1.37 (0.97–1.82)	2494.02 (1952.29–3034.19)	2.47 (1.93–3.00)	2.00 (1.88–2.13)
Central Sub-Saharan Africa	111.78 (64.38–185.27)	0.54 (0.31–0.89)	384.89 (241.15–621.24)	0.71 (0.45–1.15)	0.82 (0.65–0.99)
East Asia	4129.22 (2918.24–5559.41)	0.73 (0.52–0.98)	4290.08 (2901.56–5833.14)	0.90 (0.61–1.22)	0.65 (0.51–0.78)
Eastern Europe	1196.03 (853.92–1555.84)	1.39 (1.00–1.81)	1528.92 (1098.59–2029.11)	2.31 (1.66–3.07)	1.54 (1.32–1.76)
Eastern Sub-Saharan Africa	353.71 (252.50–478.08)	0.50 (0.36–0.67)	998.22 (721.04–1325.66)	0.57 (0.41–0.76)	0.36 (0.17–0.55)
High-income Asia Pacific	538.30 (355.80–765.60)	0.80 (0.53–1.13)	403.56 (263.05–575.67)	0.80 (0.52–1.14)	0.17 (0.00–0.34)
High-income North America	795.56 (513.30–1172.76)	0.70 (0.45–1.03)	750.50 (479.91–1089.40)	0.61 (0.39–0.88)	−0.83 (−1.05– −0.61)
North Africa and Middle East	1426.83 (1053.09–1898.60)	1.07 (0.79–1.42)	5066.51 (3787.84–6525.48)	1.99 (1.49–2.57)	1.94 (1.78–2.11)
Oceania	34.35 (22.91–49.25)	1.29 (0.86–1.85)	88.06 (55.30–131.07)	1.56 (0.98–2.33)	0.46 (0.39–0.53)
South Asia	4771.23 (3661.63–6220.77)	1.11 (0.85–1.44)	9697.32 (7461.57–12466.76)	1.23 (0.94–1.58)	0.32 (0.21–0.44)
Southeast Asia	2102.79 (1583.84–2788.74)	1.07 (0.80–1.42)	4361.25 (3405.00–5453.09)	1.57 (1.23–1.97)	1.16 (1.11–1.21)
Southern Latin America	95.63 (52.81–155.68)	0.50 (0.28–0.82)	160.01 (87.46–254.83)	0.62 (0.34–0.99)	0.81 (0.75–0.86)
Southern Sub-Saharan Africa	264.40 (194.27–349.67)	1.22 (0.90–1.62)	540.14 (410.62–700.61)	1.59 (1.21–2.06)	0.64 (0.41–0.88)
Tropical Latin America	725.19 (523.39–984.73)	1.13 (0.81–1.53)	1324.60 (989.45–1750.26)	1.50 (1.12–1.98)	0.74 (0.67–0.80)
Western Europe	552.26 (344.34–842.23)	0.38 (0.24–0.58)	535.97 (330.53–821.33)	0.41 (0.25–0.63)	0.15 (−0.00–0.30)
Western Sub-Saharan Africa	637.53 (472.40–836.87)	0.89 (0.66–1.17)	2155.30 (1645.30–2725.83)	1.13 (0.86–1.43)	0.75 (0.64–0.86)

ASIR, Age-standardized incidence rate; EAPC, Estimated annual percentage change; CI, Confidence interval; UI, Uncertainty interval.

#### Mortality

Over the past 30 years, the global number of deaths among young people attributed to CKD caused by hypertension has risen significantly, increasing from 11,294 in 1990 to 16,306 in 2021, which represents a 30.73% increase. Similarly, the ASDR rose from 0.52 per 100,000 people (95% UI, 0.38–0.68) in 1990 to 0.55 per 100,000 people (95% UI, 0.39–0.74) in 2021, with an EAPC of −0.02 (95% CI, −0.13 to 0.10). In both 1990 and 2021, the number and rate of deaths were positively correlated with age, and the incidence was higher among men than women, particularly in the age group of 34–39(2021, men and women; 87.90 vs.54.81 per 100,000 people) (as shown in Table 2, Figure 1B, Supplementary Figure 1B).

**Table 2:** Death cases and ASDR of CKD due to hypertension in 1990 and 2021, and temporal trends.

	1990		2021		1990–2021
	Death cases (95% UI)	ASDR (per 100,000 population)	Death cases (95% UI)	ASDR (per 100,000 population)	EAPCs of ASR (95% CI)
Global	11293.77 (8368.72–14947.73)	0.52 (0.38–0.68)	16306.00 (11671.08–21874.25)	0.55 (0.39–0.74)	−0.02 (−0.13, 0.10)
Sex					
Male	6584.21 (4660.43–8955.33)	0.59 (0.42–0.81)	10253.98 (7156.28–13760.02)	0.68 (0.47–0.91)	0.28 (0.19, 0.37)
Female	4709.57 (3413.75–6259.68)	0.43 (0.32–0.58)	6052.02 (4365.80–8433.59)	0.41 (0.30–0.58)	−0.47 (−0.63, -0.30)
Socio-demographic index					
Low SDI	895.97 (608.92–1279.93)	0.49 (0.33–0.69)	2164.32 (1433.07–3142.18)	0.48 (0.32–0.70)	−0.10 (−0.15, -0.05)
Low-middle SDI	2657.05 (1900.91–3556.48)	0.59 (0.42–0.78)	4753.46 (3331.05–6488.08)	0.59 (0.42–0.81)	−0.12 (−0.21, -0.03)
Middle SDI	5841.96 (4268.52–7721.31)	0.78 (0.57–1.03)	7423.19 (5272.64–9851.86)	0.80 (0.57–1.06)	−0.17 (−0.31, -0.03)
High-middle SDI	1507.18 (1073.54–2100.18)	0.33 (0.24–0.46)	1211.39 (833.90–1674.44)	0.28 (0.19–0.38)	−1.10 (−1.31, -0.88)
High SDI	380.93 (269.37–525.20)	0.11 (0.08–0.15)	736.65 (528.64–974.34)	0.21 (0.15–0.28)	2.63 (2.28, 2.98)
Region					
Andean Latin America	68.19 (45.63–98.71)	0.44 (0.30–0.64)	112.11 (72.14–168.13)	0.41 (0.27–0.62)	−0.59 (−0.95, -0.23)
Australasia	0.41 (0.31–0.53)	0.01 (0.00–0.01)	0.85 (0.52–1.32)	0.01 (0.00–0.01)	1.98 (1.62, 2.35)
Caribbean	75.33 (53.21–105.35)	0.51 (0.36–0.71)	143.43 (94.27–211.02)	0.79 (0.52–1.16)	1.76 (1.61, 1.92)
Central Asia	10.48 (6.49–16.09)	0.04 (0.02–0.06)	23.98 (14.58–37.11)	0.06 (0.04–0.10)	0.75 (0.19, 1.31)
Central Europe	47.25 (31.20–69.50)	0.10 (0.07–0.15)	18.83 (12.21–28.17)	0.05 (0.03–0.08)	−1.20 (−1.55, -0.84)
Central Latin America	177.00 (122.49–250.37)	0.26 (0.18–0.37)	470.76 (305.82–691.40)	0.47 (0.30–0.68)	2.24 (2.09, 2.39)
Central Sub-Saharan Africa	128.82 (80.18–186.32)	0.62 (0.39–0.90)	351.95 (219.11–544.68)	0.65 (0.41–1.01)	0.10 (−0.00, 0.20)
East Asia	3134.09 (2176.37–4416.75)	0.55 (0.38–0.78)	1638.04 (1036.79–2425.94)	0.34 (0.22–0.51)	−2.35 (−2.69, -2.00)
Eastern Europe	80.08 (52.01–117.10)	0.09 (0.06–0.14)	37.28 (23.17–55.48)	0.06 (0.04–0.08)	−2.84 (−3.29, -2.40)
Eastern Sub-Saharan Africa	314.54 (212.22–455.11)	0.44 (0.30–0.64)	646.60 (433.32–949.34)	0.37 (0.25–0.54)	−0.86 (−0.97, -0.75)
High-income Asia Pacific	61.01 (47.38–76.48)	0.09 (0.07–0.11)	16.83 (10.94–24.61)	0.03 (0.02–0.05)	−2.94 (−3.26, -2.61)
High-income North America	120.41 (77.73–176.17)	0.11 (0.07–0.16)	365.51 (292.74–439.70)	0.30 (0.24–0.36)	3.92 (3.58, 4.26)
North Africa and Middle East	507.63 (329.45–793.98)	0.38 (0.25–0.59)	1239.83 (797.86–1778.33)	0.49 (0.31–0.70)	1.08 (0.86, 1.30)
Oceania	7.23 (3.48–11.93)	0.27 (0.13–0.45)	19.37 (11.77–30.03)	0.34 (0.21–0.53)	0.58 (0.45, 0.70)
South Asia	1160.64 (752.16–1709.92)	0.27 (0.17–0.40)	2429.31 (1555.26–3571.69)	0.31 (0.20–0.45)	0.40 (0.25, 0.55)
Southeast Asia	4399.77 (3155.32–5662.98)	2.23 (1.60–2.87)	6617.12 (4702.91–8726.02)	2.39 (1.70–3.15)	0.06 (−0.05, 0.17)
Southern Latin America	49.65 (33.66–70.31)	0.26 (0.18–0.37)	46.45 (30.49–68.42)	0.18 (0.12–0.27)	−1.03 (−1.16, -0.90)
Southern Sub-Saharan Africa	146.04 (97.77–198.05)	0.68 (0.45–0.92)	315.30 (209.87–439.79)	0.93 (0.62–1.29)	0.13 (−0.67, 0.94)
Tropical Latin America	244.33 (166.68–350.15)	0.38 (0.26–0.54)	226.81 (150.99–318.94)	0.26 (0.17–0.36)	−1.54 (−1.81, -1.27)
Western Europe	34.57 (22.73–50.80)	0.02 (0.02–0.04)	24.38 (16.58–35.33)	0.02 (0.01–0.03)	−0.61 (−0.74, -0.48)
Western Sub-Saharan Africa	526.31 (343.63–761.59)	0.74 (0.48–1.06)	1561.27 (999.87–2266.31)	0.82 (0.52–1.19)	0.27 (0.14, 0.40)

ASDR, Age-standardized death rate; EAPC, Estimated annual percentage change; CI, Confidence interval; UI, Uncertainty interval.

#### DALYs

From 1990 to 2021, the number of DALYs associated with CKD due to hypertension among young people globally rose from 825,628 to 1,180,453, reflecting an increase of 30.06%. The EAPC was −0.01(95% CI, −0.10 to 0.08). Similarly, the ASR for DALYs due to CKD caused by hypertension increased from 37.67 per 100,000 people (95% UI, 29.51 to 48.10) to 39.68 per 100,000 people (95% UI, 29.92 to 51.22). As previously noted, the number and rate of CKD due to hypertension related DALYs increase with age, with the lowest rates observed in individuals aged 15–19 years and the highest in those aged 34–39 years in1990 and 2021. And men are still larger than women. It is noteworthy that the number and rate of DALYs related to CKD due to hypertension among individuals aged 20 to 24 have significantly increased in comparison to the preceding age group (42.64%) (as shown in Table 3, Figure 1C, Supplementary Figure 1C).

**Table 3:** Cases and ASR of DALYs to CKD due to hypertension in 1990 and 2021, and temporal trends.

	1990		2021		1990–2021
	DALYs cases (95% UI)	ASR of DALYs (per 100,000 population)	DALYs cases (95% UI)	ASR of DALYs (per 100,000 population)	EAPCs of DALYs ASR (95% CI)
Global	825628.28 (646823.70–1054246.62)	37.67 (29.51–48.10)	1180452.47 (889946.51–1523802.55)	39.68 (29.92–51.22)	−0.01 (−0.10, 0.08)
Sex					
Male	472982.28 (355838.29–617744.87)	42.67 (32.10–55.73)	722968.88 (546177.43–938620.63)	47.89 (36.18–62.17)	0.23 (0.16, 0.30)
Female	352646.00 (274227.54–447229.91)	32.55 (25.31–41.28)	457483.59 (348946.48–600116.68)	31.22 (23.82–40.96)	−0.37 (−0.49, -0.24)
Socio-demographic index					
Low SDI	64537.59 (47761.32–86845.40)	35.02 (25.91–47.12)	155837.43 (111994.54–216748.67)	34.70 (24.94–48.27)	−0.10 (−0.15, -0.06)
Low-middle SDI	193147.87 (145968.63–250264.60)	42.60 (32.19–55.20)	346844.41 (263273.31–446303.86)	43.22 (32.81–55.61)	−0.07 (−0.15, -0.00)
Middle SDI	408147.27 (316073.11–521019.26)	54.23 (42.00–69.23)	513204.04 (385123.27–660124.24)	55.33 (41.52–71.17)	−0.17 (−0.28, -0.05)
High-middle SDI	116421.04 (89820.85–150807.70)	25.73 (19.85–33.32)	96460.70 (73083.09–126929.79)	21.91 (16.60–28.83)	−0.86 (−1.00, -0.71)
High SDI	42600.37 (33177.18–55088.17)	12.28 (9.56–15.88)	66934.87 (52098.60–84186.79)	18.95 (14.75–23.83)	1.68 (1.48, 1.88)
Region					
Andean Latin America	4940.49 (3719.36–6658.55)	31.95 (24.05–43.06)	7994.47 (5773.89–11090.77)	29.52 (21.32–40.96)	−0.57 (−0.86, -0.28)
Australasia	258.24 (153.22–406.66)	3.17 (1.88–4.99)	388.58 (233.97–625.79)	3.71 (2.23–5.98)	0.13 (−0.00, 0.27)
Caribbean	5377.99 (4047.29–7190.16)	36.18 (27.23–48.37)	9680.84 (6938.39–13493.08)	53.18 (38.12–74.13)	1.53 (1.39, 1.67)
Central Asia	3431.05 (2317.51–5020.78)	12.06 (8.14–17.65)	5607.66 (3863.76–8039.09)	15.00 (10.33–21.50)	0.40 (0.28, 0.52)
Central Europe	5323.07 (3966.80–7043.71)	11.36 (8.47–15.04)	2822.50 (2071.50–3829.20)	8.06 (5.92–10.93)	−0.62 (−0.83, -0.41)
Central Latin America	15271.68 (11627.76–20345.15)	22.37 (17.03–29.80)	35702.45 (26267.92–49214.05)	35.29 (25.97–48.65)	1.76 (1.63, 1.88)
Central Sub-Saharan Africa	9020.70 (6115.84–12450.08)	43.45 (29.46–59.97)	24335.07 (16720.83–36500.25)	44.98 (30.91–67.47)	0.05 (−0.04, 0.13)
East Asia	221888.30 (165171.70–298720.78)	39.22 (29.20–52.80)	119918.18 (85919.49–166234.13)	25.03 (17.94–34.70)	−2.09 (−2.37, -1.82)
Eastern Europe	8404.81 (6341.32–11385.75)	9.80 (7.39–13.27)	4798.29 (3481.30–6693.94)	7.25 (5.26–10.12)	−1.72 (−1.98, -1.46)
Eastern Sub-Saharan Africa	21944.79 (15695.23–30583.40)	30.96 (22.14–43.14)	45330.38 (32036.05–62788.96)	25.88 (18.29–35.84)	−0.81 (−0.90, -0.72)
High-income Asia Pacific	5623.45 (4586.42–6993.98)	8.33 (6.80–10.36)	2641.93 (1920.39–3695.04)	5.23 (3.80–7.31)	−1.11 (−1.35, -0.88)
High-income North America	15499.29 (11438.34–20684.20)	13.68 (10.09–18.25)	32742.22 (26759.27–39106.33)	26.58 (21.72–31.75)	2.45 (2.24, 2.66)
North Africa and Middle East	38959.06 (29211.67–56317.94)	29.11 (21.83–42.08)	90079.24 (64601.79–120219.56)	35.43 (25.41–47.28)	0.89 (0.73, 1.05)
Oceania	646.14 (399.79–951.64)	24.32 (15.05–35.82)	1677.26 (1150.86–2377.30)	29.77 (20.43–42.19)	0.49 (0.39, 0.58)
South Asia	101461.40 (76712.38–136440.67)	23.51 (17.77–31.61)	214030.91 (157856.07–287532.55)	27.06 (19.96–36.35)	0.48 (0.34, 0.62)
Southeast Asia	288990.44 (209368.54–369320.00)	146.69 (106.28–187.47)	425188.84 (312381.38–549748.66)	153.32 (112.64–198.23)	−0.01 (−0.11, 0.09)
Southern Latin America	3959.59 (3003.88–5277.50)	20.75 (15.74–27.66)	4160.81 (3120.76–5537.88)	16.13 (12.10–21.47)	−0.58 (−0.67, -0.49)
Southern Sub-Saharan Africa	9803.32 (6965.58–12716.37)	45.35 (32.23–58.83)	20337.88 (14204.97–27690.75)	59.75 (41.74–81.36)	0.09 (−0.62, 0.82)
Tropical Latin America	18488.12 (14135.60–24650.47)	28.75 (21.98–38.33)	18530.35 (13991.34–24835.29)	20.98 (15.84–28.12)	−1.22 (−1.41, -1.02)
Western Europe	10852.52 (7448.02–15124.09)	7.53 (5.17–10.49)	9688.13 (6508.75–13983.34)	7.47 (5.02–10.78)	−0.09 (−0.17, -0.02)
Western Sub-Saharan Africa	35483.80 (24791.69–49899.86)	49.58 (34.64–69.72)	104796.45 (71304.08–148913.35)	54.81 (37.29–77.88)	0.26 (0.14, 0.38)

DALYs, Disability-Adjusted Life Years; ASR, Age-standardized rate; EAPC, Estimated annual percentage change; CI, Confidence interval; UI, Uncertainty interval.

### CKD due to hypertension in adolescents and young adults: SDI regional trends

#### Incidence

**Figure 2:**
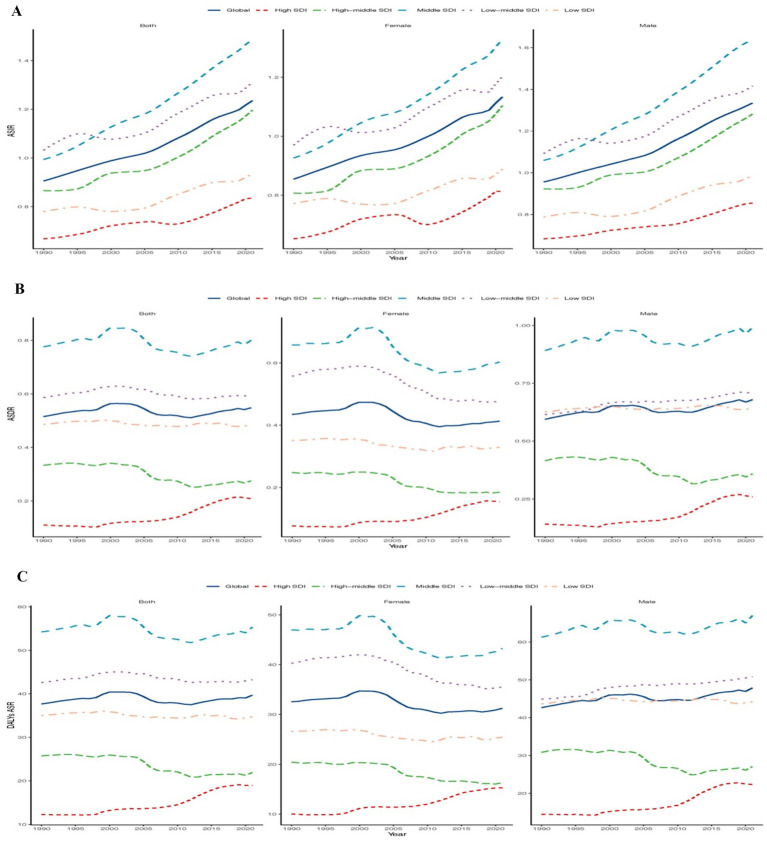
Epidemiologic trends of incidence, death, and disability-adjusted life-years (DALYs) ASR in 5 Sociodemographic Index (SDI) Regions of CKD due hypertension among adolescents and young adults From 1990 to 2021. **(A)** Trends in ASIR. **(B)** Trends in ASDR. **(C)** Trends in DALYs ASR.

In both 1990(7479.31; 95%UI, 5628.56–9731.26) and 2021(13788.87;95% UI, 10675.16–17317.09), the middle SDI experienced the highest incidence of CKD attributed to hypertension among young individuals, with EAPC of 1.31 (95% CI, 1.28–1.34). However, the ASIR presents a different trend. In 1990, the highest ASDR was 1.03 per 100,000 people (95%UI, 0.78–1.33) in the Low-middle SDI region. Furthermore, when compared to global level, the ASDR among young people is notably higher in both the Low-middle and Middle SDI region. In various SDI regions, the ASIR for both males and females exhibited an upward trend from 1990 to 2021(as shown in Table 1 and Figure 2A).

#### Mortality

In both 1990 and 2021, the middle SDI region had the highest number of CKD due to hypertension among adolescents and young adults, with a case of 5841.96 (95%UI,4268.52–7721.31) and 7423.19 (95%UI,5272.64–9851.86). Over the past thirty years, the EAPC for the high SDI region was 2.63 (95% CI, 2.28 to 2.98). Additionally, the ASDR for low-middle SDI and middle SDI regions is higher than the global average. In the low -middle and low SID regions, the ASDR among women is comparable to global levels and exhibits an upward trend. In contrast, the ASDR for men showed a downward trend in the low-middle and high-middle SID regions (as shown in Table 2 and Figure 2B).

#### DALYs

In 2021, the highest number of CKD-related DALYs due to hypertension occurred in the middle SDI region (513204.04; 95%UI, 385123.27–660124.24), while the high SDI region exhibited the lowest ASR of DALYs (18.95 per 100,000 people, 95%UI, 14.75–23.83). Notably, from 1990 to 2021, the high SDI region experienced the largest EAPC (1.68, 95% CI, 1.48 to 1.88). Furthermore, trends in DALY ASR and ASDR among both men and women were largely consistent (as shown in Table 3 and Figure 2C).

### CKD due to hypertension in adolescents and young adults: geographic regional trends

#### Incidence

**Figure 3:**
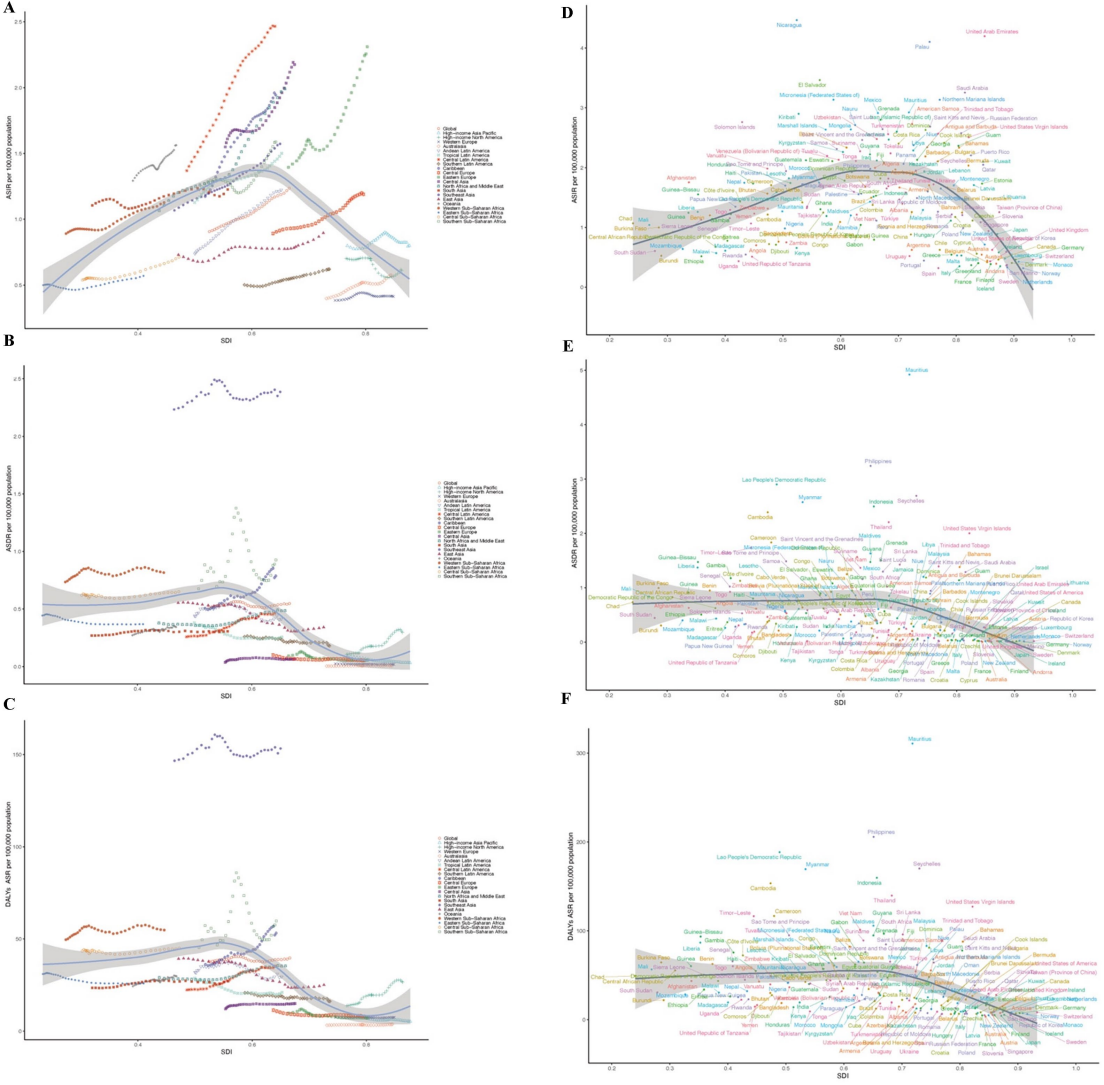
Incidence, death, and disability-adjusted life-years (DALYs) rates for CKD due hypertension among adolescents and young adults From 1990 to 2021. **(A,D)** ASIR. **(B,E)** ASDR. **(C,F)** DALYs ASR.

Among the 21 regions, South Asia recorded the highest incidence of CKD due to hypertension in both 1990 and 2021, with figures of 4,771 and 9,697, respectively. Central Asia (SDI, 0.55) had the highest ASIR in 1990(1.55 per 100,000 people; 95%UI, 1.05–2.24), while Central Latin America (SDI, 0.64) is projected to take the lead by 2021(2.47 per 100,000 people; 95%UI, 1.93–3.00). Additionally, from 1990 to 2021, Central Latin America remains the region with the greatest EACP (2.00; 95% CI, 1.88–2.13). The global SDI was 0.66 in 2021; In 1990, there were 10 regions (e.g., Central Asia, Oceania) with ASIR related to CKD caused by hypertension that exceeded the global average. By 2021, this number had decreased to 9 regions (e.g., Central Latin America, Caribbean) with ASIR higher than the global average, whereas 12 regions (eg, Western Europe and East Asia) had lower ASIR than the global mean (1.24) (as shown in Table 1 and Figure 3A).

#### Mortality

In 2021, the number of adult deaths related to CKD resulting from hypertension was the highest in Southeast Asia (6617.12; 95% UI, 4702.91–8726.02), a trend that was also observed in 1990. The region (SDI, 0.64) recorded the greatest ASDR associated with CKD (2.39; 95% UI, 1.70–3.15). In contrast, Australasia (SDI, 0.84) experienced the lowest CKD-related ASDR (0.01; 95% UI, 0.00–0.01). High-income North America saw the most substantial rise in ASDR among adolescents and young adults (EAPC, 3.92; 95% CI, 3.58 to 4.26), while High-income Asia Pacific reported the smallest decline for this age group (EAPC, −2.94; 95% CI, −3.26 to −2.61). As previously mentioned, the global SDI was 0.66 in 2021. Six regions exhibited higher CKD-related ASDR than the global average, whereas 15 regions showed lower rates than the global mean (0.55) (as shown in Table 2 and Figure 3B).

#### DALYs

Over the past three decades, as with mortality, the highest number of CKD-related DALYs was found in South-East Asia, with an EACP of −0.01 (−0.11, 0.09). In contrast, Australasia had the lowest number (388.58; 95% UI, 233.97–625.79). The region (SDI, 0.64) recorded the greatest DALYs ASR associated with CKD due to hypertension (153.32; 95% UI, 112.64–198.23). In contrast, Australasia (SDI, 0.84) experienced the lowest CKD-related DALYs ASR (3.71; 95% UI, 2.23–5.98). From 1990 to 2021, High-income North America is the greatest increase in 21 GBD region (EAPC, 2.45; 95% CI, 2.24 to 2.66), Conversely, East Asia is the smallest decrease (EAPC, −2.09; 95% CI, −2.37 to −1.82). The global SDI was 0.66 in 2021: Five regions (e.g., Caribbean, Central Sub-Saharan Africa) exhibited higher CKD-related ASR than the global average, whereas 16 regions (e.g., Central Asia, Western Europe) showed lower rates than the global mean (39.68) (as shown in Table 3 and Figure 3C).

### CKD due to hypertension in adolescents and young adults: national trends

#### Incidence

**Figure 4:**
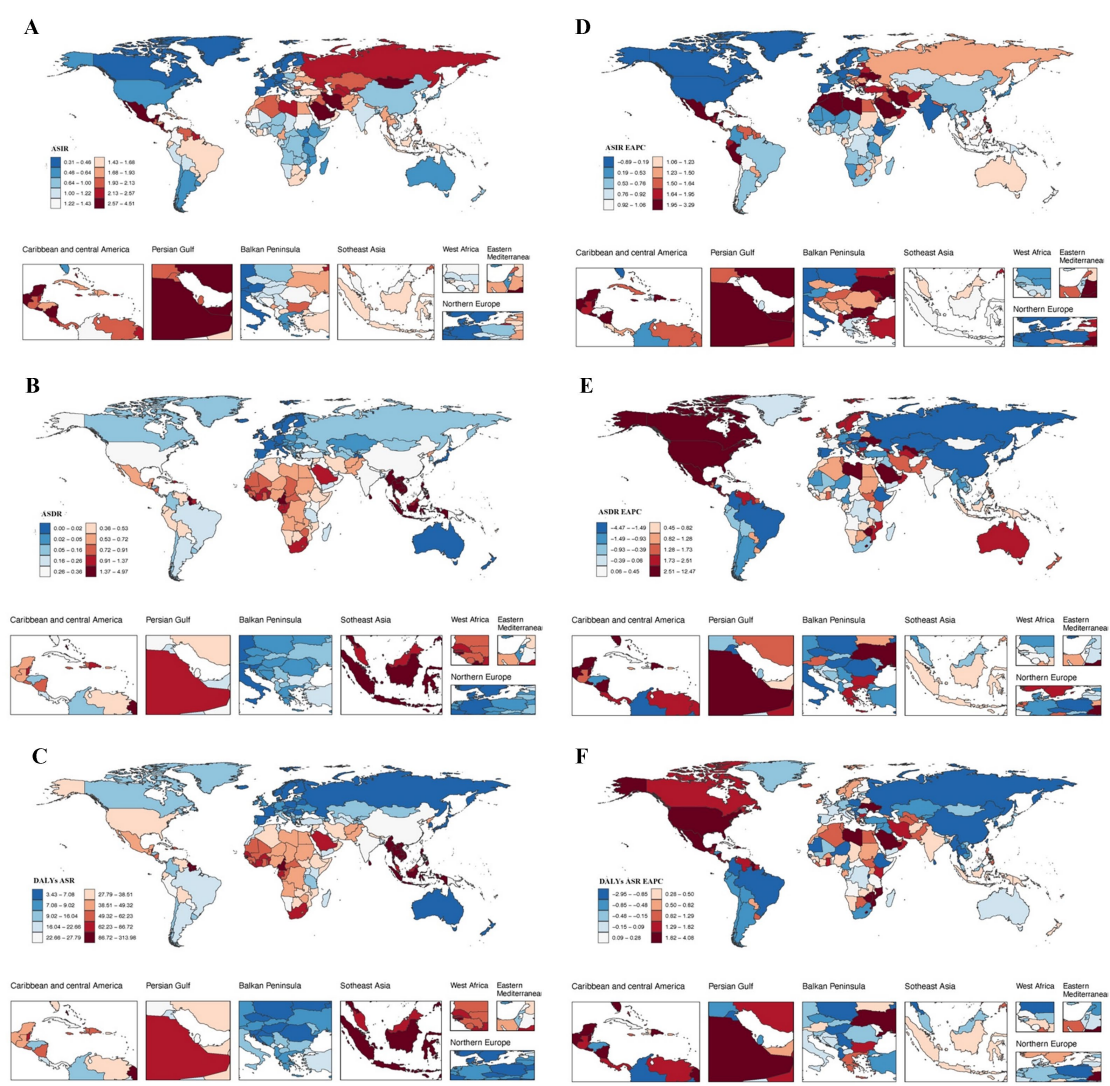
Incidence, death, and disability-adjusted life-years (DALYs) for CKD due hypertension among adolescents and young adults in 204 Countries and Territories. **(A)** ASIR. **(B)** ASDR. **(C)** DALYs ASR. **(D)** ASIR EAPC. **(E)** ASDR EAPC. **(F)** DALYs ASR EAPC.

In 2021, among 204 countries, India had the most cases of adolescents and young adults CKD due to hypertension (7252.91; 95% UI, 5597.70–9267.35); Nicaragua (SDI, 0.52) had the highest ASIR of CKD (4.47 per 100,000 population; 95% UI, 2.48–7.31), whereas Spain (SDI, 0.77) had the smallest ASIR (0.32 per 100,000 population; 95% UI, 0.15–0.57) (Figure 4A; Supplementary Table 1). The global average ASIR was 1.21 per 100,000 population (95% UI, 0.96–1.56), with incidences exceeding the global mean in 120 countries and falling below it in 84 countries (Figure 4A; Supplementary Table 1). From 1990 to 2021, the country with the highest EAPC was El Salvador, with an EAPC of 3.26 (95% CI, 3.06–3.46), while Poland had the lowest (−0.01; 95% CI, −0.27 to 0.24) (Figure 4D; Supplementary Table 1). The global ASIR-related CKD caused by hypertension among young people was 0.55 (95% UI, 0.39–0.74) in 2021. The ASIR were above the global mean in 121 countries and below the global mean in 83 countries (Figure 4A).

#### Mortality

In 2021, Indonesia reported the highest number of chronic kidney disease (CKD)-related deaths among young people due to hypertension, totaling 16,306 (95% UI, 11671.08–21874.25). Bangladesh exhibited the highest CKD-related ASDR from hypertension in this demographic, with a rate of 1.16 per 100,000 population (95% CI, 0.51–1.70), while Finland recorded the lowest ASDR at 0.00 per 100,000 population (95% CI, 0.00–0.00) (Table 2; Figure 4B). Armenia experienced the most significant increase in ASDR, with an EAPC of 4.87 (95% CI, 3.43–6.32), whereas the Russian Federation (EAPC, −4.43; 95% CI, −4.94 to −3.93) and the Republic of Korea (EAPC, −3.75; 95% CI, −4.09 to −3.41) reported the largest decreases (Supplementary Table 2; Figure 4E). In 2021, Mauritius (SDI, 0.72) had the highest ASDR related to CKD caused by hypertension, while Finland (SDI, 0.86) had the lowest ASDR. The global ASDR related to CKD caused by hypertension among young people was 0.55 (95% UI, 0.39–0.74) in 2021. The ASDR were above the global mean in 77 countries and below the global mean in 124 countries, 3 countries were at the same level (Figure 4B).

#### DALYs

In 2021, Indonesia reported the highest number of DALYs attributed to CKD caused by hypertension among adolescents and young adults (181919.81; 95% UI, 123,869.67 to 253,286.52) (Supplementary Table 3). In contrast, Mauritius exhibited the highest ASR of CKD due to hypertension DALYs within the same age group (310.88 per 100,000 population; 95% UI, 123 to 395.83) (Supplementary Table 3; Figure 4C). From 1990 to 2021, the country with the highest EAPC was Ukraine, with an EAPC of 12.35 (95% CI, 10.54–14.20).the Russian Federation (EAPC, −4.43; 95% CI, −4.94 to −3.93) and Republic of Korea (EAPC, −3.75; 95% CI, −4.09 to −3.41) had the greatest decreases (Supplementary Table 3; Figure 4F). Mauritius (SDI, 0.72) reported the highest ASR of DALYs associated with CKD due to hypertension among youth and young adults. In contrast, Australia (SDI, 0.84) recorded the lowest ASR of DALYs. In 2021, the global ASR for CKD-related DALYs was 39.68 (95% UI, 29.92–51.22). Notably, 82 countries reported ASRs exceeding the global average, while 122 countries fell below this mean (Supplementary Table 3; Figure 4C).

### Risk factors for CKD due to hypertension in adolescents and young adults

**Figure 5:**
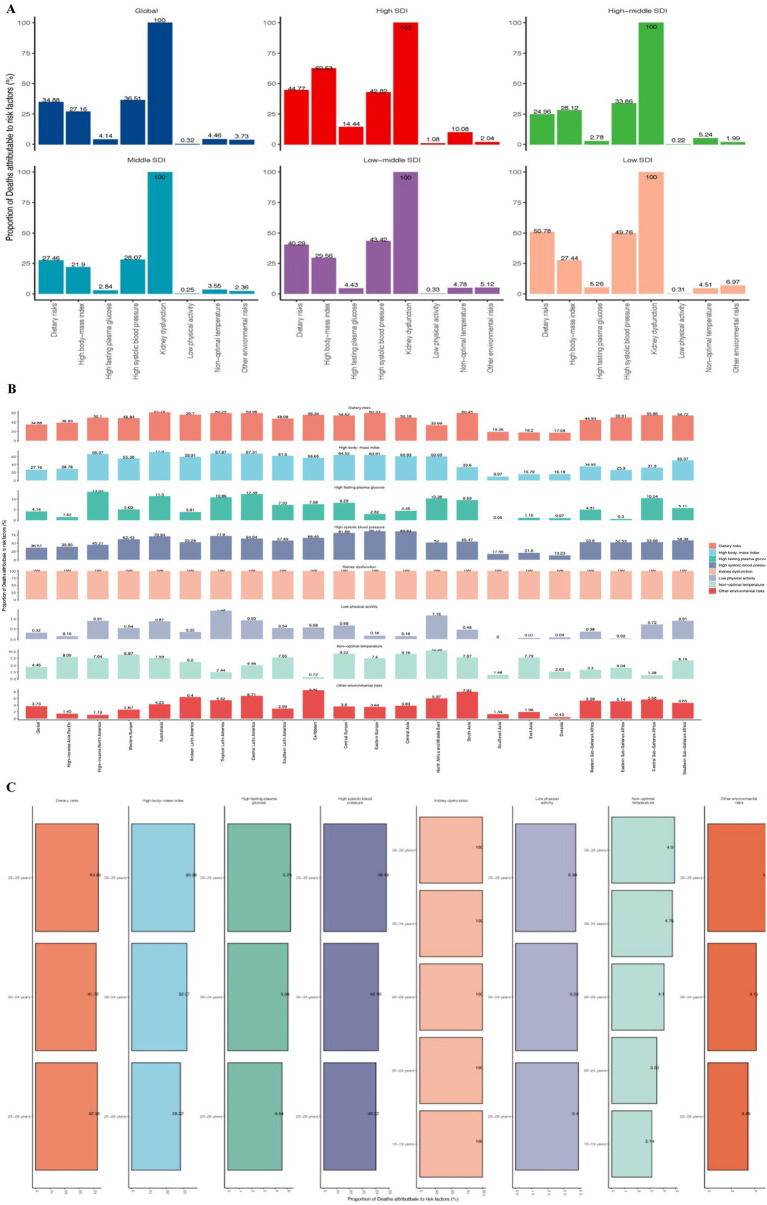
Mortality risk factors for CKD due hypertension among adolescents and young adults. **(A)** SDI regional trends. **(B)** Geographic regional trends. **(C)** Age trend.

According to the 2021 GBD database, there are eight mortality risk factors for CKD due to hypertension in adolescents and young adults: high fasting plasma glucose, dietary risks, low physical activity, non-optimal temperature, kidney dysfunction, high systolic blood pressure, high body mass index, and other environmental risks. Across both global and all 21 GBD regions (Figures 5A,B), kidney dysfunction is identified as the highest mortality risk factor for CKD due to hypertension (100%), while low physical activity represents the lowest mortality risk (e.g., low SDI regions 0.31%, Southeast Asia 0%). Furthermore, in the SDI regions, the mortality risks with the highest proportions are high body mass index, dietary risks, and high fasting plasma glucose (e.g., High SDI region; 44.77, 62.53, 42.82%). Conversely, low physical activity, non-optimal temperature, and high fasting plasma glucose account for the lowest proportions in the low and low-middle SDI regions (Figure 5A).

Mortality risk factors for CKD due to hypertension in adolescents and young adults exhibit varying proportions across 21 geographical regions (Figure 5B). Kidney dysfunction is consistently observed at the highest proportion in all countries (100%). The regions with the lowest proportions of high systolic blood pressure and dietary risks are Oceania, East Asia, and Southeast Asia. Non-optimal temperature represents the lowest proportion in the Caribbean (0.72%), while high body mass index has the highest proportion in Australasia (71.5%). Tropical Latin America has the highest proportion of low physical activity (1.39%), whereas Southeast Asia accounted for none. Other environmental risks are least represented in Oceania (0.43%). High-taste plasma glucose was highest in high-income North America (13.55%), Central Latin America (12.39%) and Australasia (11.5%), and lowest in Southeast Asia (0.05%), Oceania (0.97%) and Eastern Sub-Saharan Africa (0.5%).

Among individuals aged 15 to 39 years, only kidney dysfunction and non-optimal temperature were identified as risk factors for death from CKD due to hypertension. Within this population, the mortality risk proportion associated with kidney dysfunction is 100%. The age group with the highest mortality risk related to non-optimal temperature is 35 to 39 years, while the group with the lowest risk is 15 to 19 years (Figure 5C).

### Predictive analysis for CKD due to hypertension in adolescents and young adults

**Figure 6:**
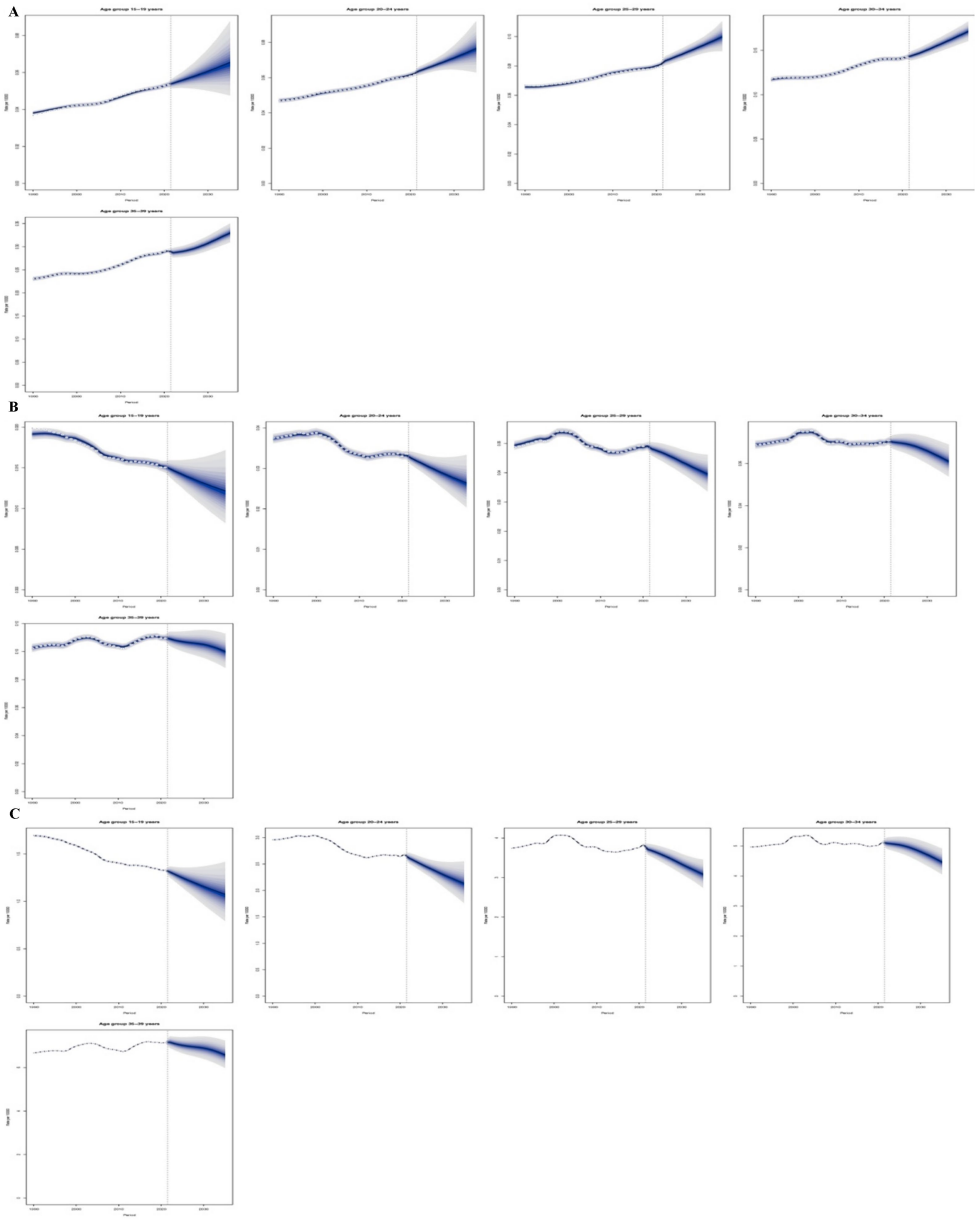
Predictive analysis for CKD due to hypertension in adolescents and young adults. **(A)** ASIR; **(B)** ASDR; **(C)** DALYs ASR.

The forecast analysis projects that by 2035, the global ASIR of CKD caused by hypertension will rise significantly among adolescents and young adults (24–29 years; 19.90%). Meanwhile, the global ASDR and DALYs ASR are exhibiting a downward trend, the most significant decrease was 20.48% (20–24 age group), while the smallest decrease was 8.42% in the 35–39 age group. Similarly, the DALYs ASR shows the most substantial decrease was 20.52% in the 20–24 age group, whereas the least decrease was 7.98% in the 35–39 age group (Figures 6A–C).

## Discussion

Over the past 30 years, there has been a notable increase in the prevalence of CKD attributed to hypertension among adolescents and young adults worldwide. This issue not only escalates healthcare and societal costs but also presents a significant challenge to global public health. The present study examined the GBD regions and nations from 1990 to 2021, focusing on the incidence, mortality, DALYs and of CKD due to hypertension in individuals aged 15 to 39. Additionally, potential risk factors were assessed, and trends were predicted for 2035. The results provide essential insights into the impact of hypertension-related CKD among those aged 15 to 39 across different income levels in various regions and countries. Furthermore, the research reinforced the analysis of data spanning from 1990 to 2021, emphasizing the increasing burden of CKD associated with hypertension in specific areas worldwide. A comprehensive evaluation of the disease’s epidemiological trends will aid healthcare professionals in developing effective management strategies.

This research extensively examined the condition of CKD due to hypertension among adolescents and young adults, leading to several key findings: First, from 1990 to 2021, the ASIR of CKD due to hypertension among global adolescents and young adults increased significantly. The ASDR and the DALYs have shown relative stability after an initial decline. Second, in 2021, geographic region or countries with a medium SDI exhibited the highest levels of ASIR, ASDR, and DALYs, with a notable acceleration in the ASIR growth trend. In contrast, high SDI geographic region or countries recorded the lowest levels for these three indicators, consequently, regardless of geographic region or countries, the ASR of these three indicators is positively correlated with underdeveloped countries and negatively correlated with developed countries. Third, global data indicates that across different age groups, the ASIR, ASDR, and DALYs for males are higher than those for females. Additionally, the burden of CKD attributable to hypertension is positively correlated with age. Fourth, the primary risk factors for CKD caused by hypertension among adolescents and young adults include elevated blood glucose levels, dietary risks, high blood pressure, and increased body mass index. These risk factors exhibit varying distribution patterns across different regions, age groups, and genders. Finally, looking ahead to 2035, the ASIR of CKD among global adolescents and young adults is expected to rise significantly, while ASDR and DALYs may show a downward trend, with variations depending on specific age groups.

Hypertension is recognized as a significant death of risk factor for CKD and is strongly endorsed by expert consensus (12). While the mechanisms of CKD due to hypertension are relatively well understood, the underlying factors are influenced by various metabolic and environmental determinants (10, 12). Variations in these underlying factors are associated with regional and age-related differences in the incidence and mortality of CKD attributable to hypertension. Consequently, further investigation into these risk factors presents a promising approach to preventing CKD induced by hypertension in adolescents and young adults. This study, therefore, integrates secondary-level risk factors from the 2021 GBD database and identifies eight risk factors associated with CKD due to hypertension: high fasting plasma glucose, dietary risks, low physical activity, non-optimal temperature, kidney dysfunction, high systolic blood pressure, high body mass index, and other environmental risks. Our research indicates that kidney dysfunction is the primary mortality risk factor for the development of CKD due to hypertension among young individuals across the country. This issue is not limited to specific age groups or regions. Additionally, at the level of SDI Region, high systolic blood pressure, dietary risks, and high body mass index are the most significant mortality risk factors for CKD among adolescents and young adults, consistent with existing literature (6, 19). At the national level, the risk factors in Oceania and Southeast Asia are among the lowest. This may be attributed to the ongoing improvements in diagnostic methods, medical care services, and the management and treatment of hypertension in these regions (20, 21). Our analysis also supports these findings. In addition to global renal dysfunction, our study indicate non-optimal temperature is a significant risk factor for individuals aged 15–39 with CKD due to hypertension. Non-optimal temperatures can directly or indirectly harm the kidneys through various physiological pathways, including changes in blood flow dynamics, oxidative stress, and fluctuations in blood pressure (22–25). Consequently, controlling environmental temperatures and avoiding extreme exposure are critical measures for the prevention of CKD due to hypertension, especially for high-risk groups that require enhanced protective strategies. This study found that the primary risk factors for death are predominantly concentrated in the age group of 25–39 with CKD due to hypertension. As age increases, the proportion of these factors shows a positive correlation, which is consistent with the report (26). This further indicates that the internal risk factors have gradually supplanted external factors as the core contributors to the primary risk of death. Consequently, the secondary prevention of risk factors, along with the screening for hypertension in high-risk groups and the provision of high-quality medical services, can reduce the burden of CKD caused by hypertension, ultimately leading to improved health outcomes for patients (27, 28).

Our analysis identifies hypertension as a key modifiable driver of CKD in young adults, though globally, diabetes remains the primary cause. Disease burden patterns vary regionally: hypertension-CKD rises with urbanization and NCD transitions, potentially surpassing infection-related CKD in low-SDI areas. Hypertension amplifies future cardiovascular risk. Projections show rising ASIR but falling ASDR and DALY rates by 2035, indicating increased survival with disability. Central SDI regions face significant challenges. Integrating BP screening into youth diabetes/obesity programs and implementing cost-effective strategies (e.g., telehealth monitoring, salt reduction) in moderate-SDI areas are critical.

This study is the first to predict and analyze the burden of CKD attributable to hypertension from 2022 to 2035, offering valuable insights for the formulation of long-term health investment strategies and policy planning. The results indicate that, although the global ASIR of hypertension-related CKD is anticipated to continue rising, both the ASDR and the DALYs ASR are expected to decline overall. Notably, this trend is most pronounced among individuals aged 20 to 24. The increase in the ASIR of CKD due to hypertension among the youth population is primarily attributed to a surge in lifestyle-related risk factors and improved diagnostic capabilities. In contrast, the decline in ASDR and DALYs ASR is dependent on advancements in medical technology. Nevertheless, the long-term burden of disability, such as dependence on dialysis, may continue to rise in this demographic. It is crucial to disrupt the vicious cycle of “hypertension-chronic kidney disease (CKD)-cardiovascular incidents” through early intervention.

### Limitations

This study presents both innovations and limitations. Firstly, existing research predominantly relies on the previous GBD2019 database and lacks a thorough analysis of CKD due to hypertension, particularly concerning adolescents and young population. Secondly, all data is sourced from the GBD database, which may compromise accuracy due to the varying availability of registered data across different countries. Thirdly, regional differences may lead to diverse diagnostic methods for hypertension and CKD, influenced by various factors such as laboratory examination techniques and the methods used for calculating estimated glomerular filtration rate (eGFR). Finally, the information regarding adolescents and young population is also constrained by the limited understanding of other risk factors for death associated with CKD due to hypertension.

## Conclusion

Over the last three decades, the burden of CKD attributable to hypertension among adolescents and the young adult population has steadily risen worldwide, and projections suggest this trend will persist through 2035. Results from this cross-sectional analysis demonstrate that, while the ASDR and DALYs ASR related to CKD due to hypertension among adolescents and young individuals show only slight variation globally, there has been a significant rise in the EAPC in the ASIR pertinent to this issue. Furthermore, in middle SDI region, CKD-related ASDR and DALYs ASR associated with hypertension among adolescents and young population have reached highest levels. Presently, factors such as kidney dysfunction, high systolic blood pressure, and dietary risks are the leading causes of mortality associated with CKD linked to hypertension in these populations. Consequently, it is essential for the global healthcare system to formulate more personalized prevention strategies to effectively alleviate the economic impact of CKD as a result of hypertension.

## Data Availability

The datasets presented in this study can be found in online repositories. The names of the repository/repositories and accession number(s) can be found in the article/Supplementary material.

## Footnotes

^1^ http://ghdx.healthdata.org/gbd-results-tool
