# Supplementary material for: A epidemiological trend of chronic kidney disease due to hypertension among adolescents and young adults: global burden and future 2035 projections
Source: Front Public Health. 2025 Aug 4;13:1618416. doi: 10.3389/fpubh.2025.1618416 (PMC12360296; doi:10.3389/fpubh.2025.1618416)
Supplement: Supplementary file 1 [file Table_1.DOCX]

**Supplement**

**eFigure** **1:** Trends in CKD due to hypertension Incidence(a), Deaths(b), and Disability-Adjusted Life-Years (DALYs) (c) among adolescents and young adults in 1990.

**eFigure 2：** Incidence, Death, and Disability-Adjusted Life-Years (DALYs) for CKD due to hypertension among adolescents and young adults in 204 Countries and Territories in 1990. A, ASIR. B, ASDR. C, DALYs ASR.

**eTable 1:** Incidence for CKD due to hypertension among adolescents and young adults in 204 Countries and Territories in 1990 and 2021.

**eTable 2:** Death for CKD due to hypertension among adolescents and young adults in 204 Countries and Territories in 1990 and 2021.

**eTable 3:** Disability-Adjusted Life-Years (DALYs) for CKD due to hypertension among adolescents and young adults in 204 Countries and Territories in 1990 and 2021.

| 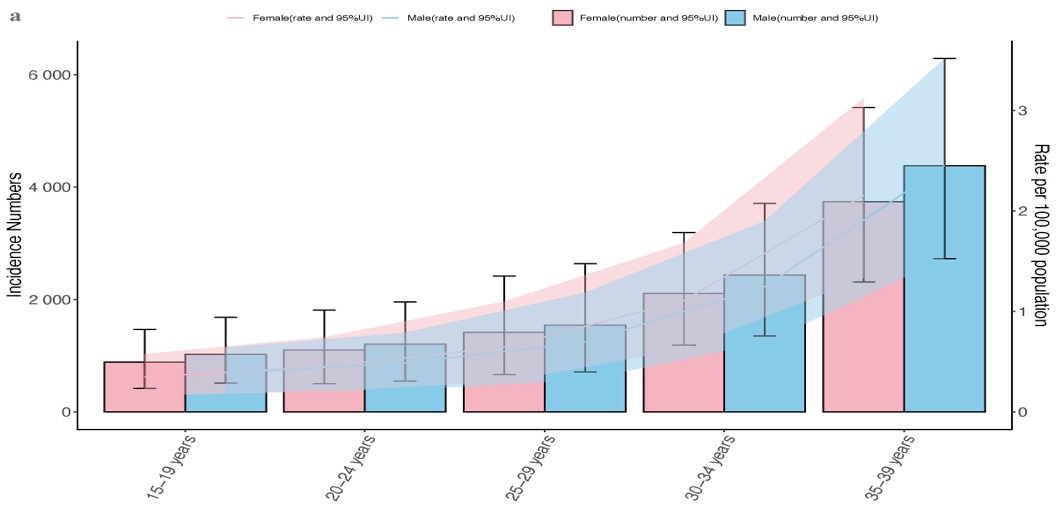 |
| --- |
|  |
|  |
| **eFigure 1:** Trends in CKD due to hypertension Incidence(a), Deaths(b), and Disability-Adjusted Life-Years (DALYs) (c) among adolescents and young adults in 1990 |

| 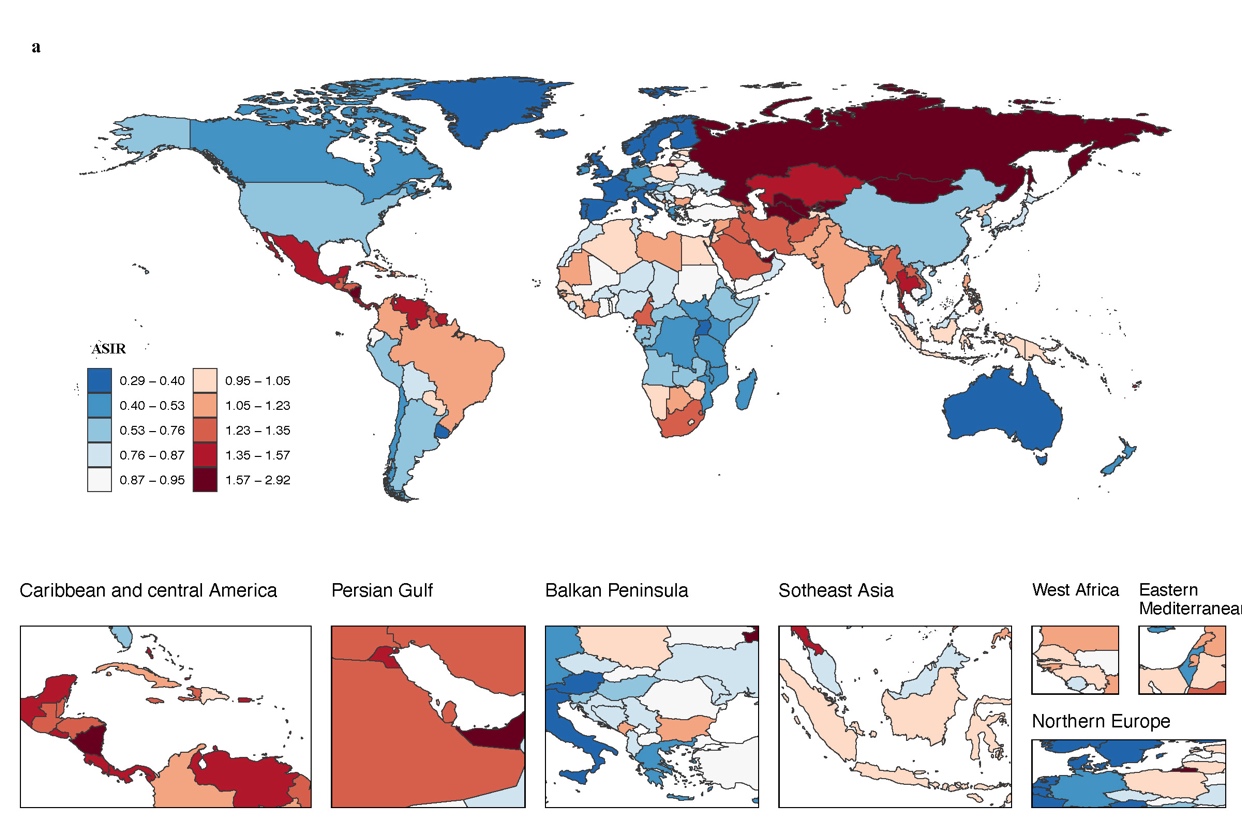 |
| --- |
|  |
| 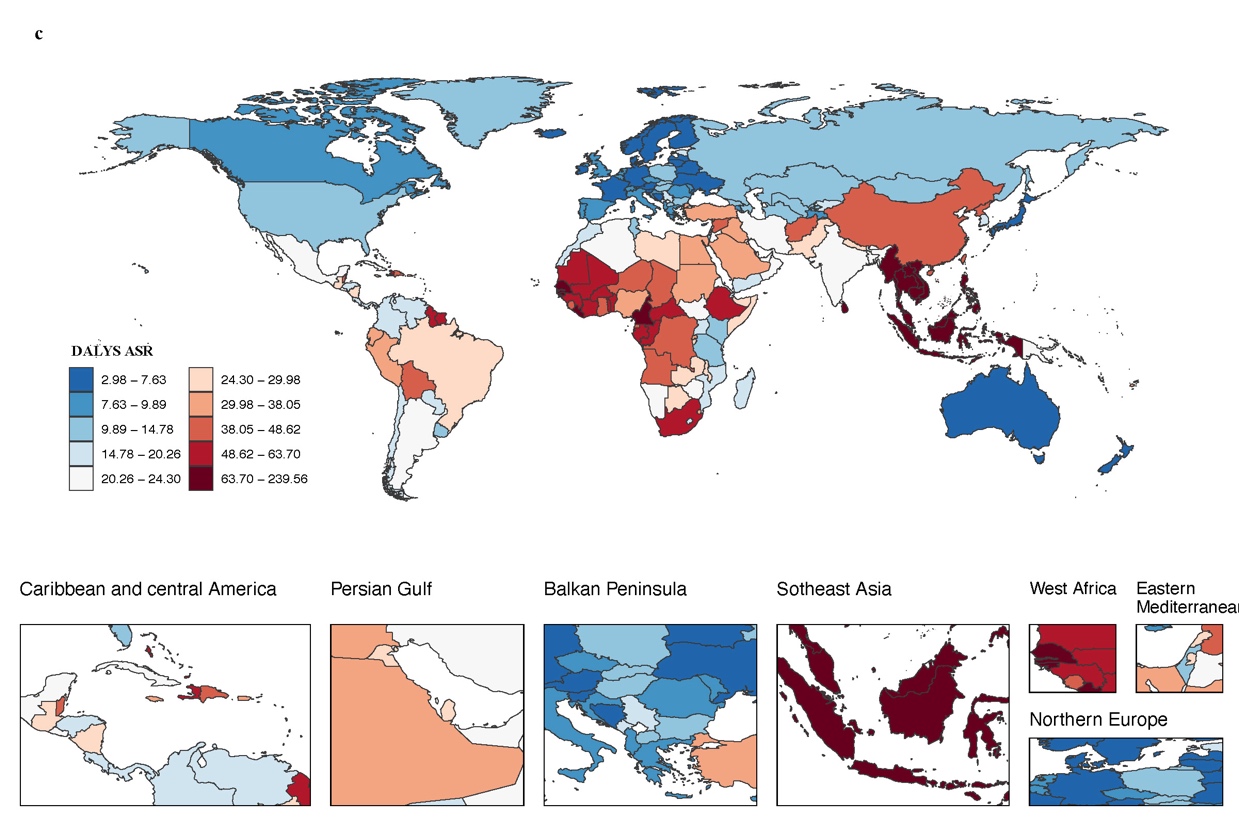 |
| **eFigure 2：**Incidence, Death, and Disability-Adjusted Life-Years (DALYs) for CKD due to hypertension among adolescents and young adults in 204 Countries and Territories in 1990. A, ASIR. B, ASDR. C, DALYs ASR. |

| **eTable 1 Incidence cases and ASIR of CKD due to hypertension in 1990 and 2021, and temporal trends in 204 Countries and Territories** | | | | | |
| --- | --- | --- | --- | --- | --- |
|  | **1990** |  | **2021** |  | **1990-2021** |
|  | **Incidence cases (95% UI)** | **ASIR (per 100,000 population)** | **Incidence cases (95% UI)** | **ASIR (per 100,000 population)** | **EAPCs of ASR(95% CI)** |
| **Afghanistan** | 38.98(18.76-75.85) | 1.24(0.60-2.41) | 214.42(106.22-404.42) | 1.75(0.87-3.31) | 1.73(1.36,2.10) |
| **Albania** | 11.53(5.79-20.99) | 0.81(0.41-1.48) | 13.23(6.65-22.92) | 1.40(0.70-2.42) | 1.74(1.57,1.91) |
| **Algeria** | 102.95(52.05-180.48) | 1.02(0.52-1.79) | 339.99(179.82-572.90) | 2.00(1.06-3.36) | 2.08(1.89,2.27) |
| **American Samoa** | 0.35(0.18-0.63) | 1.74(0.90-3.10) | 0.48(0.25-0.84) | 2.73(1.43-4.78) | 1.43(1.32,1.54) |
| **Andorra** | 0.09(0.04-0.16) | 0.34(0.17-0.62) | 0.10(0.05-0.18) | 0.39(0.20-0.72) | 0.60(0.42,0.78) |
| **Angola** | 21.16(10.70-37.80) | 0.54(0.27-0.97) | 85.37(43.40-159.78) | 0.70(0.36-1.31) | 0.75(0.52,0.98) |
| **Antigua and Barbuda** | 0.37(0.20-0.65) | 1.45(0.78-2.53) | 0.81(0.43-1.37) | 2.34(1.25-3.98) | 1.52(1.36,1.69) |
| **Argentina** | 66.14(34.43-114.45) | 0.54(0.28-0.94) | 109.55(53.05-198.80) | 0.63(0.30-1.13) | 0.70(0.58,0.81) |
| **Armenia** | 14.72(7.07-27.71) | 1.02(0.49-1.93) | 17.93(9.39-31.80) | 1.67(0.87-2.96) | 1.35(1.06,1.64) |
| **Australia** | 25.40(12.05-43.42) | 0.38(0.18-0.64) | 47.27(23.41-78.67) | 0.55(0.27-0.91) | 1.11(0.98,1.25) |
| **Austria** | 10.67(4.97-20.99) | 0.36(0.17-0.70) | 12.56(6.10-23.74) | 0.44(0.22-0.84) | 0.44(0.19,0.69) |
| **Azerbaijan** | 40.22(18.34-72.97) | 1.27(0.58-2.30) | 83.42(39.37-142.58) | 1.97(0.93-3.37) | 1.35(1.18,1.52) |
| **Bahamas** | 1.69(0.94-2.90) | 1.43(0.79-2.45) | 3.61(2.09-6.23) | 2.33(1.35-4.03) | 1.64(1.43,1.85) |
| **Bahrain** | 2.71(1.42-4.53) | 1.06(0.55-1.77) | 12.00(6.28-19.80) | 1.71(0.89-2.81) | 1.60(1.33,1.87) |
| **Bangladesh** | 206.66(102.20-374.51) | 0.49(0.24-0.89) | 524.45(269.28-942.29) | 0.76(0.39-1.37) | 1.20(0.99,1.42) |
| **Barbados** | 1.52(0.82-2.57) | 1.40(0.75-2.35) | 2.16(1.19-3.63) | 2.19(1.21-3.67) | 1.44(1.28,1.60) |
| **Belarus** | 35.80(17.95-63.66) | 0.91(0.46-1.61) | 48.92(25.32-82.94) | 1.67(0.86-2.83) | 1.74(1.30,2.17) |
| **Belgium** | 14.34(6.42-27.05) | 0.39(0.17-0.73) | 14.69(6.96-27.91) | 0.42(0.20-0.80) | 0.04(-0.11,0.19) |
| **Belize** | 0.92(0.49-1.64) | 1.26(0.67-2.25) | 4.41(2.33-7.30) | 2.34(1.23-3.87) | 2.03(1.91,2.15) |
| **Benin** | 15.63(7.75-26.98) | 0.92(0.46-1.58) | 63.01(32.23-111.97) | 1.20(0.61-2.14) | 0.85(0.76,0.94) |
| **Bermuda** | 0.31(0.17-0.52) | 1.22(0.67-2.04) | 0.36(0.19-0.59) | 2.03(1.11-3.39) | 1.69(1.50,1.88) |
| **Bhutan** | 2.65(1.34-4.71) | 0.98(0.50-1.75) | 5.43(2.80-9.27) | 1.57(0.81-2.67) | 1.50(1.39,1.62) |
| **Bolivia (Plurinational State of)** | 19.75(10.20-35.71) | 0.80(0.41-1.45) | 53.66(28.67-93.19) | 1.09(0.58-1.90) | 1.02(0.97,1.08) |
| **Bosnia and Herzegovina** | 15.34(7.84-26.37) | 0.81(0.41-1.39) | 12.64(6.71-21.55) | 1.26(0.67-2.14) | 1.38(1.25,1.51) |
| **Botswana** | 5.56(2.98-9.36) | 1.08(0.58-1.82) | 17.90(10.17-29.06) | 1.68(0.95-2.73) | 1.30(1.05,1.54) |
| **Brazil** | 709.91(511.58-965.93) | 1.13(0.82-1.54) | 1276.71(944.75-1687.69) | 1.50(1.11-1.98) | 0.72(0.65,0.79) |
| **Brunei Darussalam** | 1.13(0.55-1.93) | 0.91(0.45-1.57) | 2.55(1.27-4.38) | 1.25(0.62-2.15) | 1.01(0.91,1.10) |
| **Bulgaria** | 31.57(16.03-54.87) | 1.06(0.54-1.84) | 40.01(21.22-69.28) | 2.11(1.12-3.65) | 2.49(2.39,2.59) |
| **Burkina Faso** | 24.40(12.13-42.14) | 0.77(0.38-1.32) | 86.53(44.12-152.32) | 1.00(0.51-1.76) | 0.89(0.81,0.98) |
| **Burundi** | 11.45(5.64-20.71) | 0.55(0.27-1.00) | 27.77(13.84-50.84) | 0.53(0.26-0.96) | -0.41(-0.65,-0.17) |
| **Cabo Verde** | 0.99(0.52-1.76) | 0.75(0.40-1.35) | 3.56(1.99-6.18) | 1.42(0.79-2.46) | 1.87(1.71,2.04) |
| **Cambodia** | 34.71(17.75-64.60) | 0.90(0.46-1.68) | 88.00(46.24-154.69) | 1.21(0.64-2.14) | 0.71(0.51,0.91) |
| **Cameroon** | 49.08(23.74-86.81) | 1.29(0.62-2.28) | 220.14(110.47-381.21) | 1.71(0.86-2.96) | 0.81(0.66,0.97) |
| **Canada** | 50.35(24.34-90.61) | 0.45(0.22-0.82) | 54.92(26.94-94.46) | 0.46(0.23-0.80) | -0.14(-0.38,0.09) |
| **Central African Republic** | 6.98(3.50-12.71) | 0.67(0.34-1.22) | 20.10(10.35-36.25) | 0.92(0.47-1.66) | 0.94(0.87,1.02) |
| **Chad** | 18.25(9.28-35.11) | 0.87(0.44-1.67) | 67.24(34.70-124.65) | 1.07(0.55-1.98) | 0.62(0.45,0.79) |
| **Chile** | 24.98(12.06-46.43) | 0.44(0.21-0.81) | 44.23(21.88-78.59) | 0.63(0.31-1.11) | 1.00(0.83,1.17) |
| **China** | 3968.53(2800.46-5362.95) | 0.72(0.51-0.98) | 4069.78(2736.34-5551.01) | 0.88(0.59-1.20) | 0.62(0.48,0.76) |
| **Colombia** | 159.89(82.66-265.21) | 1.14(0.59-1.89) | 276.35(149.70-472.47) | 1.38(0.74-2.35) | 0.47(0.38,0.57) |
| **Comoros** | 1.06(0.51-1.86) | 0.62(0.30-1.08) | 2.23(1.09-3.93) | 0.72(0.35-1.27) | 0.39(0.32,0.47) |
| **Congo** | 6.80(3.33-13.16) | 0.72(0.35-1.39) | 21.57(10.86-39.40) | 0.97(0.49-1.78) | 0.95(0.83,1.07) |
| **Cook Islands** | 0.10(0.05-0.17) | 1.26(0.62-2.15) | 0.14(0.07-0.24) | 2.41(1.20-4.08) | 2.24(2.03,2.45) |
| **Costa Rica** | 20.16(10.29-35.24) | 1.57(0.80-2.74) | 45.70(25.80-77.81) | 2.40(1.36-4.09) | 1.20(1.12,1.27) |
| **Côte d'Ivoire** | 51.65(23.69-98.31) | 1.09(0.50-2.08) | 160.77(81.09-290.00) | 1.43(0.72-2.59) | 0.79(0.63,0.94) |
| **Croatia** | 14.70(7.34-26.58) | 0.81(0.40-1.46) | 14.66(7.72-25.04) | 1.17(0.62-2.01) | 1.25(1.21,1.29) |
| **Cuba** | 51.33(27.42-89.82) | 1.05(0.56-1.84) | 65.24(36.53-109.24) | 1.82(1.02-3.05) | 1.62(1.25,2.00) |
| **Cyprus** | 1.24(0.61-2.24) | 0.40(0.20-0.73) | 2.81(1.43-4.99) | 0.56(0.29-0.99) | 0.89(0.80,0.98) |
| **Czechia** | 29.40(16.26-49.86) | 0.79(0.44-1.34) | 32.94(18.60-55.96) | 1.12(0.63-1.90) | 1.34(1.21,1.46) |
| **Democratic People's Republic of Korea** | 79.41(43.19-136.76) | 0.95(0.52-1.64) | 125.59(68.07-208.67) | 1.25(0.68-2.07) | 0.76(0.60,0.92) |
| **Democratic Republic of the Congo** | 73.42(37.03-138.72) | 0.51(0.26-0.97) | 244.52(125.71-444.61) | 0.68(0.35-1.23) | 0.83(0.65,1.00) |
| **Denmark** | 7.42(3.43-13.80) | 0.39(0.18-0.72) | 7.41(3.65-13.45) | 0.41(0.20-0.74) | 0.10(-0.16,0.35) |
| **Djibouti** | 0.87(0.42-1.55) | 0.50(0.24-0.88) | 3.84(1.97-6.52) | 0.71(0.36-1.21) | 1.04(0.83,1.25) |
| **Dominica** | 0.52(0.27-0.91) | 1.77(0.94-3.11) | 0.68(0.37-1.19) | 2.63(1.44-4.59) | 1.07(0.90,1.25) |
| **Dominican Republic** | 29.84(15.74-52.52) | 0.97(0.51-1.71) | 87.62(47.48-149.68) | 1.93(1.04-3.29) | 2.34(2.22,2.47) |
| **Ecuador** | 37.48(18.13-66.85) | 0.91(0.44-1.62) | 111.81(58.65-189.46) | 1.53(0.80-2.59) | 1.81(1.75,1.87) |
| **Egypt** | 228.65(123.41-367.24) | 1.04(0.56-1.68) | 752.18(422.34-1205.64) | 1.78(1.00-2.86) | 1.61(1.40,1.83) |
| **El Salvador** | 30.60(16.41-55.44) | 1.46(0.79-2.65) | 89.79(47.64-160.53) | 3.46(1.84-6.19) | 3.26(3.06,3.46) |
| **Equatorial Guinea** | 0.78(0.40-1.42) | 0.52(0.26-0.94) | 5.53(2.77-9.88) | 0.79(0.40-1.42) | 1.61(1.42,1.80) |
| **Eritrea** | 8.35(4.08-16.15) | 0.64(0.31-1.25) | 21.76(11.21-40.45) | 0.78(0.40-1.44) | 0.41(0.28,0.53) |
| **Estonia** | 5.75(2.77-10.18) | 1.01(0.49-1.79) | 6.85(3.48-12.03) | 1.73(0.88-3.04) | 1.68(1.49,1.87) |
| **Eswatini** | 3.87(1.88-6.73) | 1.28(0.62-2.23) | 10.73(5.42-18.13) | 2.11(1.07-3.56) | 1.39(1.14,1.64) |
| **Ethiopia** | 100.75(67.57-155.35) | 0.55(0.37-0.85) | 240.04(157.26-359.87) | 0.52(0.34-0.78) | -0.35(-0.56,-0.13) |
| **Fiji** | 5.03(2.64-8.54) | 1.56(0.82-2.65) | 7.43(4.11-12.42) | 2.08(1.15-3.48) | 0.73(0.61,0.84) |
| **Finland** | 5.30(2.46-9.61) | 0.29(0.14-0.53) | 6.44(3.08-11.35) | 0.39(0.18-0.68) | 0.46(0.26,0.65) |
| **France** | 79.20(37.25-144.03) | 0.36(0.17-0.65) | 80.29(37.84-142.22) | 0.40(0.19-0.72) | 0.22(0.11,0.32) |
| **Gabon** | 2.64(1.34-5.06) | 0.69(0.35-1.32) | 7.81(3.83-14.72) | 1.04(0.51-1.96) | 1.22(1.09,1.36) |
| **Gambia** | 3.24(1.59-6.10) | 0.86(0.42-1.62) | 12.21(6.03-21.81) | 1.22(0.60-2.18) | 1.11(0.99,1.22) |
| **Georgia** | 27.62(14.40-46.90) | 1.30(0.68-2.20) | 24.17(13.42-40.67) | 2.13(1.18-3.58) | 1.63(1.47,1.80) |
| **Germany** | 133.39(66.72-249.11) | 0.45(0.22-0.84) | 116.81(59.71-220.74) | 0.46(0.24-0.87) | -0.20(-0.45,0.06) |
| **Ghana** | 51.24(25.41-87.84) | 0.89(0.44-1.53) | 186.54(95.68-321.65) | 1.30(0.67-2.25) | 1.14(1.04,1.24) |
| **Global** | 19849.19(14719.58-25805.45) | 0.91(0.67-1.18) | 36753.78(28504.14-46380.72) | 1.24(0.96-1.56) | 0.99(0.95,1.03) |
| **Greece** | 16.90(7.88-31.04) | 0.45(0.21-0.83) | 15.18(7.26-26.21) | 0.55(0.26-0.94) | 0.83(0.68,0.99) |
| **Greenland** | 0.08(0.04-0.15) | 0.31(0.15-0.56) | 0.08(0.04-0.13) | 0.37(0.19-0.63) | -0.08(-0.40,0.23) |
| **Grenada** | 0.55(0.30-0.93) | 1.64(0.89-2.78) | 1.13(0.62-1.90) | 2.80(1.54-4.71) | 1.68(1.58,1.78) |
| **Guam** | 0.90(0.50-1.57) | 1.42(0.79-2.47) | 1.31(0.73-2.27) | 2.36(1.32-4.10) | 1.55(1.35,1.74) |
| **Guatemala** | 39.73(20.65-67.38) | 1.34(0.70-2.28) | 144.53(74.88-249.05) | 2.12(1.10-3.66) | 1.65(1.60,1.70) |
| **Guinea** | 19.48(9.61-34.86) | 0.95(0.47-1.70) | 64.29(30.90-118.06) | 1.24(0.60-2.28) | 0.80(0.70,0.91) |
| **Guinea-Bissau** | 4.55(2.19-8.51) | 1.23(0.59-2.30) | 13.10(6.38-24.05) | 1.55(0.76-2.85) | 0.69(0.58,0.81) |
| **Guyana** | 4.59(2.42-7.78) | 1.35(0.71-2.29) | 6.90(3.68-11.59) | 2.22(1.18-3.73) | 1.52(1.34,1.70) |
| **Haiti** | 32.70(17.13-55.80) | 1.34(0.70-2.29) | 95.14(50.54-157.18) | 1.73(0.92-2.86) | 0.87(0.76,0.99) |
| **Honduras** | 23.28(12.74-40.13) | 1.35(0.74-2.32) | 76.58(42.62-129.49) | 1.74(0.97-2.95) | 0.93(0.87,0.99) |
| **Hungary** | 27.38(13.48-47.82) | 0.74(0.36-1.29) | 30.64(15.30-51.55) | 1.11(0.56-1.87) | 1.64(1.52,1.75) |
| **Iceland** | 0.35(0.17-0.63) | 0.33(0.16-0.61) | 0.42(0.20-0.78) | 0.35(0.17-0.65) | 0.08(-0.01,0.17) |
| **India** | 4040.32(3111.53-5260.13) | 1.18(0.91-1.54) | 7252.91(5597.70-9267.35) | 1.19(0.92-1.52) | 0.05(-0.08,0.18) |
| **Indonesia** | 817.05(596.08-1088.58) | 1.05(0.76-1.39) | 1723.56(1321.40-2185.04) | 1.51(1.16-1.92) | 1.05(0.99,1.11) |
| **Iran (Islamic Republic of)** | 274.33(198.18-367.03) | 1.26(0.91-1.69) | 902.41(654.91-1196.06) | 2.60(1.89-3.45) | 2.02(1.76,2.28) |
| **Iraq** | 96.88(49.66-165.96) | 1.35(0.69-2.31) | 369.32(193.20-614.01) | 2.12(1.11-3.52) | 1.63(1.49,1.76) |
| **Ireland** | 7.31(3.30-13.16) | 0.53(0.24-0.96) | 9.11(4.35-15.29) | 0.58(0.28-0.98) | 0.53(0.48,0.59) |
| **Israel** | 8.35(4.22-14.58) | 0.44(0.22-0.76) | 17.64(9.15-30.99) | 0.53(0.28-0.93) | 0.87(0.77,0.96) |
| **Italy** | 75.01(44.36-119.44) | 0.35(0.21-0.56) | 55.33(33.43-87.50) | 0.35(0.21-0.55) | 0.03(-0.18,0.24) |
| **Jamaica** | 13.21(7.11-23.24) | 1.34(0.72-2.36) | 29.46(15.89-49.47) | 2.47(1.33-4.15) | 1.98(1.77,2.19) |
| **Japan** | 379.95(255.20-532.34) | 0.85(0.57-1.19) | 288.24(188.89-408.56) | 0.89(0.58-1.26) | 0.34(0.18,0.50) |
| **Jordan** | 16.11(8.83-27.94) | 1.05(0.57-1.82) | 101.19(57.05-173.30) | 1.88(1.06-3.23) | 1.97(1.91,2.04) |
| **Kazakhstan** | 102.22(52.61-188.45) | 1.51(0.77-2.78) | 143.69(76.95-253.80) | 2.06(1.10-3.64) | 0.89(0.63,1.15) |
| **Kenya** | 38.51(27.52-51.06) | 0.44(0.31-0.58) | 142.23(105.90-186.70) | 0.66(0.49-0.86) | 1.13(0.97,1.28) |
| **Kiribati** | 0.65(0.34-1.16) | 2.13(1.11-3.79) | 1.44(0.75-2.54) | 2.90(1.50-5.10) | 0.92(0.86,0.98) |
| **Kuwait** | 12.53(6.68-20.69) | 1.48(0.79-2.45) | 44.52(24.37-75.32) | 2.10(1.15-3.55) | 1.04(0.88,1.20) |
| **Kyrgyzstan** | 35.02(17.30-66.51) | 1.94(0.96-3.69) | 61.56(31.57-110.48) | 2.26(1.16-4.06) | 0.30(0.16,0.45) |
| **Lao People's Democratic Republic** | 19.96(10.23-36.41) | 1.29(0.66-2.36) | 50.97(27.22-89.80) | 1.59(0.85-2.80) | 0.49(0.32,0.66) |
| **Latvia** | 8.75(4.44-14.48) | 0.92(0.47-1.52) | 9.20(4.88-15.06) | 1.71(0.91-2.80) | 1.78(1.50,2.06) |
| **Lebanon** | 12.66(6.96-21.42) | 1.10(0.60-1.86) | 44.98(24.24-74.17) | 1.94(1.04-3.20) | 1.56(1.41,1.71) |
| **Lesotho** | 5.55(2.83-9.26) | 1.03(0.52-1.72) | 15.16(7.72-25.59) | 1.82(0.93-3.08) | 1.82(1.57,2.06) |
| **Liberia** | 8.15(4.12-14.41) | 0.88(0.45-1.56) | 28.29(14.43-49.58) | 1.26(0.64-2.21) | 1.22(1.05,1.40) |
| **Libya** | 18.56(9.54-30.92) | 1.10(0.57-1.84) | 68.64(37.35-111.74) | 2.29(1.24-3.72) | 2.42(2.28,2.55) |
| **Lithuania** | 14.02(7.06-26.62) | 1.01(0.51-1.91) | 11.83(5.99-21.60) | 1.47(0.74-2.68) | 0.94(0.71,1.18) |
| **Luxembourg** | 0.60(0.29-1.05) | 0.41(0.19-0.71) | 1.06(0.49-1.90) | 0.48(0.22-0.86) | 0.49(0.28,0.70) |
| **Madagascar** | 22.88(11.10-40.77) | 0.51(0.24-0.90) | 71.64(35.62-129.45) | 0.61(0.30-1.11) | 0.59(0.46,0.71) |
| **Malawi** | 16.83(8.30-30.48) | 0.45(0.22-0.82) | 46.69(22.74-86.13) | 0.57(0.28-1.05) | 0.86(0.67,1.04) |
| **Malaysia** | 64.36(32.77-116.10) | 0.87(0.44-1.56) | 179.68(97.81-301.72) | 1.29(0.70-2.17) | 1.08(0.98,1.19) |
| **Maldives** | 0.74(0.35-1.35) | 0.91(0.43-1.66) | 3.72(1.86-6.31) | 1.43(0.72-2.43) | 1.03(0.78,1.28) |
| **Mali** | 26.54(13.05-46.07) | 0.89(0.44-1.54) | 92.72(47.19-161.60) | 1.04(0.53-1.81) | 0.44(0.35,0.54) |
| **Malta** | 0.55(0.27-0.97) | 0.40(0.19-0.70) | 0.71(0.35-1.24) | 0.53(0.26-0.93) | 0.95(0.84,1.07) |
| **Marshall Islands** | 0.30(0.16-0.55) | 1.76(0.92-3.19) | 0.63(0.34-1.14) | 2.64(1.43-4.80) | 1.35(1.26,1.44) |
| **Mauritania** | 8.20(4.07-14.88) | 1.07(0.53-1.94) | 21.65(10.75-38.59) | 1.27(0.63-2.26) | 0.45(0.35,0.55) |
| **Mauritius** | 8.58(4.40-15.11) | 1.73(0.89-3.04) | 13.31(7.04-23.69) | 2.92(1.55-5.20) | 1.76(1.69,1.83) |
| **Mexico** | 507.69(367.18-675.92) | 1.42(1.03-1.90) | 1500.33(1184.94-1848.28) | 2.91(2.30-3.59) | 2.46(2.28,2.65) |
| **Micronesia (Federated States of)** | 0.82(0.42-1.49) | 2.04(1.04-3.72) | 1.33(0.69-2.37) | 3.14(1.63-5.58) | 1.41(1.34,1.49) |
| **Monaco** | 0.04(0.02-0.07) | 0.39(0.20-0.71) | 0.04(0.02-0.07) | 0.43(0.21-0.77) | 0.20(-0.03,0.42) |
| **Mongolia** | 15.68(7.68-28.61) | 1.77(0.87-3.24) | 33.31(18.39-55.78) | 2.64(1.46-4.42) | 1.06(0.97,1.15) |
| **Montenegro** | 3.10(1.68-5.45) | 1.23(0.67-2.17) | 3.50(1.88-6.16) | 1.70(0.91-3.00) | 1.13(1.06,1.20) |
| **Morocco** | 88.84(47.98-151.12) | 0.85(0.46-1.45) | 256.63(140.35-435.22) | 1.75(0.96-2.96) | 2.34(2.15,2.53) |
| **Mozambique** | 22.20(10.81-39.97) | 0.47(0.23-0.84) | 76.95(37.48-136.92) | 0.64(0.31-1.14) | 1.10(0.96,1.23) |
| **Myanmar** | 230.17(116.45-404.22) | 1.34(0.68-2.35) | 384.92(195.74-664.16) | 1.71(0.87-2.95) | 0.70(0.62,0.78) |
| **Namibia** | 5.67(3.01-9.97) | 1.01(0.54-1.78) | 12.55(6.68-21.89) | 1.20(0.64-2.09) | 0.40(0.19,0.62) |
| **Nauru** | 0.08(0.04-0.14) | 1.96(0.99-3.40) | 0.13(0.06-0.23) | 2.71(1.35-4.85) | 0.99(0.92,1.05) |
| **Nepal** | 69.09(35.85-120.20) | 0.95(0.49-1.65) | 234.14(120.61-411.52) | 1.75(0.90-3.07) | 1.62(1.51,1.73) |
| **Netherlands** | 18.13(7.95-35.33) | 0.30(0.13-0.59) | 17.57(8.49-32.06) | 0.33(0.16-0.61) | 0.15(-0.08,0.38) |
| **New Zealand** | 7.07(3.59-12.01) | 0.51(0.26-0.87) | 12.85(7.00-21.37) | 0.71(0.39-1.19) | 0.94(0.76,1.13) |
| **Nicaragua** | 30.54(16.43-51.32) | 2.07(1.11-3.48) | 127.14(70.72-208.02) | 4.47(2.48-7.31) | 2.51(2.42,2.61) |
| **Niger** | 23.81(11.58-44.22) | 0.86(0.42-1.59) | 89.06(42.58-163.87) | 1.00(0.48-1.84) | 0.51(0.45,0.57) |
| **Nigeria** | 278.98(204.34-363.99) | 0.82(0.60-1.07) | 884.28(664.97-1152.97) | 0.98(0.74-1.28) | 0.64(0.52,0.77) |
| **Niue** | 0.01(0.01-0.02) | 1.59(0.83-2.73) | 0.01(0.01-0.02) | 2.38(1.25-4.09) | 1.34(1.25,1.43) |
| **North Macedonia** | 7.10(3.68-12.78) | 0.89(0.46-1.61) | 12.25(6.46-20.85) | 1.60(0.84-2.73) | 2.01(1.90,2.12) |
| **Northern Mariana Islands** | 0.57(0.30-1.06) | 2.45(1.29-4.54) | 0.52(0.26-0.90) | 3.13(1.60-5.43) | 0.38(0.03,0.74) |
| **Norway** | 4.76(2.73-7.58) | 0.30(0.17-0.47) | 5.64(3.19-8.86) | 0.32(0.18-0.50) | 0.01(-0.14,0.17) |
| **Oman** | 6.74(3.72-11.24) | 0.81(0.45-1.35) | 38.19(20.74-64.19) | 1.65(0.90-2.77) | 1.90(1.28,2.53) |
| **Pakistan** | 452.51(296.17-684.21) | 1.11(0.73-1.68) | 1680.39(1119.47-2500.54) | 1.70(1.13-2.53) | 1.22(1.16,1.28) |
| **Palau** | 0.15(0.08-0.27) | 2.19(1.09-3.80) | 0.24(0.12-0.42) | 4.10(2.10-7.05) | 1.92(1.72,2.11) |
| **Palestine** | 8.76(4.36-15.54) | 1.14(0.57-2.03) | 33.92(17.67-59.90) | 1.55(0.81-2.74) | 0.67(0.53,0.81) |
| **Panama** | 13.77(7.22-24.23) | 1.36(0.71-2.40) | 34.06(18.40-58.07) | 2.06(1.11-3.52) | 1.37(1.23,1.51) |
| **Papua New Guinea** | 17.05(8.69-29.73) | 1.03(0.52-1.80) | 57.63(29.73-97.99) | 1.35(0.69-2.29) | 0.79(0.71,0.88) |
| **Paraguay** | 15.28(7.99-25.84) | 0.97(0.51-1.65) | 47.88(25.79-83.94) | 1.57(0.84-2.74) | 1.39(1.26,1.52) |
| **Peru** | 56.91(29.72-98.80) | 0.64(0.33-1.11) | 165.46(89.67-283.12) | 1.11(0.60-1.91) | 1.96(1.89,2.02) |
| **Philippines** | 276.86(207.42-356.42) | 1.07(0.80-1.38) | 935.19(743.35-1137.24) | 1.98(1.57-2.41) | 1.89(1.81,1.96) |
| **Poland** | 139.05(87.97-204.19) | 0.96(0.61-1.41) | 119.53(77.24-172.18) | 0.99(0.64-1.42) | -0.01(-0.27,0.24) |
| **Portugal** | 14.03(6.99-24.62) | 0.37(0.18-0.65) | 13.16(6.58-24.17) | 0.45(0.22-0.82) | 0.92(0.69,1.15) |
| **Puerto Rico** | 19.43(10.47-32.54) | 1.37(0.74-2.30) | 22.80(12.82-38.14) | 2.20(1.24-3.69) | 1.68(1.50,1.87) |
| **Qatar** | 3.01(1.54-5.24) | 1.27(0.65-2.22) | 33.84(17.76-57.36) | 2.05(1.07-3.47) | 0.85(0.50,1.19) |
| **Republic of Korea** | 144.79(75.12-241.89) | 0.69(0.36-1.15) | 94.70(50.97-160.95) | 0.59(0.32-1.01) | -0.33(-0.55,-0.10) |
| **Republic of Moldova** | 13.75(7.21-23.81) | 0.79(0.41-1.37) | 18.50(10.06-31.51) | 1.49(0.81-2.54) | 1.82(1.45,2.19) |
| **Romania** | 77.40(39.68-140.91) | 0.89(0.46-1.62) | 68.07(36.40-117.08) | 1.26(0.68-2.17) | 1.40(1.25,1.55) |
| **Russian Federation** | 964.99(699.52-1264.63) | 1.66(1.20-2.17) | 1196.47(853.64-1549.81) | 2.57(1.84-3.33) | 1.28(1.05,1.52) |
| **Rwanda** | 16.14(7.75-30.47) | 0.59(0.28-1.11) | 30.98(14.92-54.68) | 0.55(0.26-0.96) | -0.40(-0.65,-0.15) |
| **Saint Kitts and Nevis** | 0.30(0.16-0.52) | 1.76(0.94-3.00) | 0.61(0.34-0.99) | 2.69(1.52-4.35) | 1.23(1.04,1.41) |
| **Saint Lucia** | 0.80(0.41-1.35) | 1.43(0.73-2.40) | 1.75(0.92-2.98) | 2.64(1.39-4.51) | 1.95(1.77,2.14) |
| **Saint Vincent and the Grenadines** | 0.62(0.31-1.06) | 1.34(0.68-2.30) | 1.02(0.54-1.72) | 2.46(1.30-4.16) | 2.00(1.81,2.19) |
| **Samoa** | 1.02(0.54-1.80) | 1.52(0.81-2.69) | 1.86(1.00-3.17) | 2.31(1.25-3.95) | 1.29(1.17,1.41) |
| **San Marino** | 0.03(0.01-0.06) | 0.32(0.15-0.61) | 0.03(0.02-0.06) | 0.37(0.18-0.67) | 0.58(0.33,0.82) |
| **Sao Tome and Principe** | 0.52(0.26-0.97) | 1.21(0.60-2.25) | 1.77(0.91-3.29) | 1.94(1.00-3.62) | 1.64(1.54,1.74) |
| **Saudi Arabia** | 87.47(44.86-149.06) | 1.32(0.68-2.24) | 602.94(337.68-1011.03) | 3.26(1.82-5.46) | 2.74(2.52,2.96) |
| **Senegal** | 26.29(12.86-48.45) | 0.95(0.47-1.76) | 74.47(37.79-136.30) | 1.15(0.59-2.11) | 0.58(0.46,0.70) |
| **Serbia** | 30.98(16.74-51.84) | 0.86(0.47-1.44) | 36.08(19.18-61.00) | 1.22(0.65-2.06) | 1.32(1.24,1.39) |
| **Seychelles** | 0.37(0.19-0.61) | 1.17(0.59-1.94) | 0.75(0.40-1.24) | 1.95(1.05-3.24) | 1.51(1.36,1.66) |
| **Sierra Leone** | 13.62(6.40-25.02) | 0.85(0.40-1.57) | 42.12(20.36-76.82) | 1.13(0.55-2.06) | 0.79(0.63,0.96) |
| **Singapore** | 12.44(6.51-21.28) | 0.82(0.43-1.41) | 18.08(9.34-31.39) | 0.94(0.49-1.63) | 0.53(0.32,0.74) |
| **Slovakia** | 18.50(9.44-32.25) | 0.90(0.46-1.58) | 20.63(10.85-34.42) | 1.21(0.63-2.01) | 1.01(0.88,1.13) |
| **Slovenia** | 5.10(2.61-8.93) | 0.67(0.34-1.16) | 6.18(3.21-10.88) | 1.09(0.56-1.91) | 1.63(1.55,1.70) |
| **Solomon Islands** | 3.71(1.69-6.69) | 2.90(1.32-5.21) | 7.55(3.60-13.58) | 2.76(1.32-4.96) | -0.31(-0.41,-0.22) |
| **Somalia** | 18.16(8.99-34.60) | 0.63(0.31-1.19) | 56.17(28.11-104.06) | 0.68(0.34-1.26) | 0.32(0.09,0.56) |
| **South Africa** | 205.23(148.13-281.52) | 1.30(0.94-1.79) | 400.48(298.17-527.80) | 1.65(1.23-2.18) | 0.54(0.30,0.78) |
| **South Sudan** | 10.77(5.20-20.03) | 0.47(0.23-0.87) | 21.81(11.13-38.62) | 0.61(0.31-1.07) | 0.82(0.68,0.96) |
| **Spain** | 56.12(26.97-103.33) | 0.38(0.18-0.70) | 39.69(18.97-70.18) | 0.32(0.15-0.57) | 0.19(-0.07,0.46) |
| **Sri Lanka** | 76.79(41.81-130.11) | 1.04(0.57-1.76) | 118.33(63.50-204.05) | 1.47(0.79-2.53) | 1.23(1.18,1.28) |
| **Sudan** | 70.50(37.85-120.02) | 0.93(0.50-1.57) | 249.53(134.28-427.60) | 1.35(0.73-2.31) | 1.13(0.93,1.33) |
| **Suriname** | 2.20(1.13-3.86) | 1.35(0.69-2.37) | 4.88(2.56-7.98) | 2.27(1.19-3.72) | 1.64(1.47,1.80) |
| **Sweden** | 9.02(4.60-15.40) | 0.31(0.16-0.52) | 11.81(6.72-19.01) | 0.36(0.21-0.59) | 0.19(0.04,0.34) |
| **Switzerland** | 11.65(5.93-20.37) | 0.44(0.23-0.77) | 12.82(6.30-23.12) | 0.46(0.23-0.83) | -0.06(-0.22,0.10) |
| **Syrian Arab Republic** | 56.83(27.60-96.04) | 1.18(0.58-2.00) | 81.09(44.33-133.87) | 1.59(0.87-2.63) | 1.23(1.02,1.44) |
| **Taiwan (Province of China)** | 81.28(43.58-141.97) | 0.88(0.47-1.54) | 94.72(51.40-163.10) | 1.26(0.68-2.16) | 1.14(1.02,1.26) |
| **Tajikistan** | 20.24(9.55-37.22) | 0.96(0.45-1.76) | 55.24(28.02-98.05) | 1.32(0.67-2.35) | 1.10(0.91,1.30) |
| **Thailand** | 350.67(186.80-632.01) | 1.35(0.72-2.44) | 384.98(215.79-686.97) | 1.82(1.02-3.24) | 0.95(0.88,1.01) |
| **Timor-Leste** | 2.96(1.54-5.37) | 0.93(0.48-1.69) | 6.09(3.11-10.78) | 1.07(0.54-1.89) | 0.35(0.27,0.43) |
| **Togo** | 12.89(6.23-23.99) | 0.94(0.45-1.75) | 43.51(21.15-78.35) | 1.29(0.63-2.33) | 0.98(0.84,1.13) |
| **Tokelau** | 0.01(0.00-0.01) | 1.37(0.73-2.39) | 0.01(0.01-0.02) | 2.24(1.22-3.90) | 1.65(1.58,1.71) |
| **Tonga** | 0.51(0.27-0.91) | 1.38(0.73-2.47) | 0.82(0.43-1.44) | 2.11(1.12-3.70) | 1.34(1.28,1.41) |
| **Trinidad and Tobago** | 6.93(3.51-12.17) | 1.38(0.70-2.43) | 12.79(7.07-20.79) | 2.57(1.42-4.18) | 2.01(1.90,2.12) |
| **Tunisia** | 29.10(15.71-49.69) | 0.85(0.46-1.44) | 73.48(41.51-121.32) | 1.69(0.95-2.79) | 2.12(1.86,2.37) |
| **Turkey** | 212.76(114.66-348.70) | 0.89(0.48-1.46) | 504.36(268.36-859.25) | 1.58(0.84-2.70) | 1.65(1.56,1.73) |
| **Turkmenistan** | 24.75(12.23-47.85) | 1.61(0.80-3.12) | 53.23(27.68-100.38) | 2.56(1.33-4.83) | 1.62(1.53,1.71) |
| **Tuvalu** | 0.05(0.03-0.09) | 1.49(0.78-2.57) | 0.11(0.06-0.19) | 2.20(1.15-3.84) | 1.17(1.13,1.21) |
| **Uganda** | 22.88(11.48-40.51) | 0.36(0.18-0.63) | 75.00(37.27-130.86) | 0.44(0.22-0.76) | 0.59(0.40,0.78) |
| **Ukraine** | 152.97(75.67-258.35) | 0.81(0.40-1.36) | 237.15(134.39-377.59) | 1.72(0.97-2.74) | 2.63(2.51,2.76) |
| **United Arab Emirates** | 16.09(8.60-26.51) | 1.68(0.90-2.77) | 168.61(93.43-271.80) | 4.20(2.33-6.77) | 2.56(1.92,3.21) |
| **United Kingdom** | 77.33(48.02-117.08) | 0.37(0.23-0.56) | 94.17(57.83-140.28) | 0.43(0.27-0.64) | 0.19(0.02,0.36) |
| **United Republic of Tanzania** | 43.84(20.73-80.22) | 0.45(0.21-0.83) | 119.49(59.36-212.59) | 0.51(0.25-0.91) | 0.30(0.06,0.54) |
| **United States of America** | 745.11(478.03-1097.89) | 0.73(0.47-1.07) | 695.49(446.62-1010.05) | 0.62(0.40-0.91) | -0.88(-1.10,-0.66) |
| **United States Virgin Islands** | 0.59(0.30-1.03) | 1.50(0.77-2.61) | 0.58(0.31-0.99) | 2.51(1.33-4.26) | 1.66(1.53,1.79) |
| **Uruguay** | 4.50(2.28-7.85) | 0.40(0.20-0.69) | 6.23(3.16-10.95) | 0.52(0.26-0.92) | 1.00(0.96,1.05) |
| **Uzbekistan** | 160.47(80.79-316.03) | 1.87(0.94-3.68) | 340.48(174.15-637.01) | 2.48(1.27-4.64) | 0.88(0.75,1.01) |
| **Vanuatu** | 0.83(0.45-1.52) | 1.42(0.76-2.59) | 2.47(1.37-4.36) | 1.98(1.10-3.50) | 0.99(0.95,1.04) |
| **Venezuela (Bolivarian Republic of)** | 109.02(58.64-200.50) | 1.36(0.73-2.50) | 199.55(108.54-351.63) | 2.13(1.16-3.76) | 1.53(1.46,1.60) |
| **Viet Nam** | 216.53(105.63-369.36) | 0.76(0.37-1.30) | 465.68(237.25-765.42) | 1.21(0.62-1.99) | 1.58(1.49,1.66) |
| **Yemen** | 41.57(22.00-67.27) | 0.90(0.48-1.46) | 169.55(90.97-279.04) | 1.23(0.66-2.03) | 1.12(0.76,1.49) |
| **Zambia** | 18.76(9.25-36.03) | 0.62(0.30-1.19) | 60.73(30.77-112.62) | 0.75(0.38-1.39) | 0.57(0.38,0.76) |
| **Zimbabwe** | 38.53(20.44-64.55) | 0.97(0.52-1.63) | 83.31(46.62-137.34) | 1.31(0.74-2.17) | 0.79(0.59,1.00) |
| ***ASIR Age-standardized incidence rate, EAPC Estimated annual percentage change, CI Confidence interval, UI Uncertainty interval*** | | | | | |

| **eTable 2 Denth cases and ASDR of CKD due to hypertension in 1990 and 2021, and temporal trends in 204 Countries and Territories** | | | | | |
| --- | --- | --- | --- | --- | --- |
|  | **1990** |  | **2021** |  | **1990-2021** |
|  | **Death cases (95% UI)** | **ASDR (per 100,000 population)** | **Death cases (95% UI)** | **ASDR (per 100,000 population)** | **EAPCs of ASR(95% CI)** |
| **Afghanistan** | 20.44(11.90-35.01) | 0.65(0.38-1.11) | 76.87(37.31-133.76) | 0.63(0.31-1.09) | 0.45(-0.01,0.92) |
| **Albania** | 0.86(0.55-1.32) | 0.06(0.04-0.09) | 0.45(0.27-0.73) | 0.05(0.03-0.08) | -1.39(-1.76,-1.01) |
| **Algeria** | 28.62(16.81-49.11) | 0.28(0.17-0.49) | 68.39(40.12-106.51) | 0.40(0.24-0.63) | 1.17(0.88,1.47) |
| **American Samoa** | 0.05(0.03-0.09) | 0.25(0.14-0.42) | 0.12(0.06-0.20) | 0.67(0.36-1.13) | 3.29(2.92,3.66) |
| **Andorra** | 0.01(0.00-0.01) | 0.03(0.01-0.04) | 0.00(0.00-0.01) | 0.02(0.01-0.03) | -0.42(-0.70,-0.14) |
| **Angola** | 23.93(15.02-36.77) | 0.61(0.38-0.94) | 74.80(45.00-121.99) | 0.61(0.37-1.00) | 0.23(0.03,0.42) |
| **Antigua and Barbuda** | 0.19(0.13-0.26) | 0.74(0.52-1.00) | 0.27(0.19-0.37) | 0.79(0.55-1.08) | 1.63(1.02,2.24) |
| **Argentina** | 38.09(25.33-54.04) | 0.31(0.21-0.44) | 35.72(23.83-53.43) | 0.20(0.14-0.30) | -1.18(-1.33,-1.02) |
| **Armenia** | 0.09(0.06-0.15) | 0.01(0.00-0.01) | 0.35(0.20-0.56) | 0.03(0.02-0.05) | 4.87(3.43,6.32) |
| **Australia** | 0.26(0.22-0.32) | 0.00(0.00-0.00) | 0.55(0.33-0.88) | 0.01(0.00-0.01) | 2.17(1.68,2.65) |
| **Austria** | 0.66(0.43-0.97) | 0.02(0.01-0.03) | 0.68(0.51-0.89) | 0.02(0.02-0.03) | 1.42(1.03,1.82) |
| **Azerbaijan** | 1.41(0.83-2.25) | 0.04(0.03-0.07) | 2.22(1.24-3.55) | 0.05(0.03-0.08) | -0.47(-0.88,-0.05) |
| **Bahamas** | 0.91(0.65-1.29) | 0.77(0.55-1.09) | 2.14(1.36-3.07) | 1.38(0.88-1.98) | 1.97(1.78,2.16) |
| **Bahrain** | 0.68(0.39-1.22) | 0.26(0.15-0.47) | 2.86(1.74-4.32) | 0.41(0.25-0.61) | 0.84(0.43,1.25) |
| **Bangladesh** | 113.92(72.31-176.19) | 0.27(0.17-0.42) | 145.81(86.62-224.90) | 0.21(0.13-0.33) | -0.84(-1.08,-0.60) |
| **Barbados** | 0.63(0.44-0.86) | 0.57(0.40-0.78) | 0.69(0.44-1.01) | 0.70(0.45-1.02) | 0.79(0.48,1.11) |
| **Belarus** | 0.63(0.41-0.94) | 0.02(0.01-0.02) | 0.90(0.54-1.39) | 0.03(0.02-0.05) | 1.17(0.58,1.76) |
| **Belgium** | 0.95(0.61-1.40) | 0.03(0.02-0.04) | 0.87(0.55-1.28) | 0.02(0.02-0.04) | -0.05(-0.29,0.19) |
| **Belize** | 0.39(0.28-0.54) | 0.53(0.38-0.73) | 2.36(1.64-3.39) | 1.25(0.87-1.79) | 3.28(2.76,3.80) |
| **Benin** | 15.27(9.98-22.85) | 0.90(0.59-1.34) | 49.77(30.30-74.28) | 0.95(0.58-1.42) | 0.05(-0.10,0.20) |
| **Bermuda** | 0.09(0.06-0.12) | 0.34(0.24-0.48) | 0.06(0.04-0.09) | 0.36(0.22-0.52) | 0.50(0.19,0.81) |
| **Bhutan** | 0.79(0.41-1.27) | 0.29(0.15-0.47) | 1.29(0.72-2.15) | 0.37(0.21-0.62) | 0.41(0.30,0.52) |
| **Bolivia (Plurinational State of)** | 13.45(8.12-20.90) | 0.55(0.33-0.85) | 24.72(13.92-40.42) | 0.50(0.28-0.82) | -0.72(-0.97,-0.47) |
| **Bosnia and Herzegovina** | 0.98(0.63-1.46) | 0.05(0.03-0.08) | 0.47(0.28-0.74) | 0.05(0.03-0.07) | -0.48(-1.37,0.42) |
| **Botswana** | 1.82(0.94-3.54) | 0.35(0.18-0.69) | 5.07(2.70-9.95) | 0.48(0.25-0.93) | 0.92(0.64,1.20) |
| **Brazil** | 240.80(164.30-345.15) | 0.38(0.26-0.55) | 217.77(144.08-307.20) | 0.26(0.17-0.36) | -1.60(-1.87,-1.33) |
| **Brunei Darussalam** | 0.34(0.21-0.52) | 0.27(0.17-0.42) | 0.51(0.31-0.79) | 0.25(0.15-0.39) | -0.39(-0.70,-0.07) |
| **Bulgaria** | 2.89(1.82-4.38) | 0.10(0.06-0.15) | 3.01(1.74-5.06) | 0.16(0.09-0.27) | 2.32(1.87,2.76) |
| **Burkina Faso** | 27.08(17.23-39.18) | 0.85(0.54-1.23) | 86.13(53.32-127.68) | 1.00(0.62-1.48) | 0.68(0.48,0.88) |
| **Burundi** | 7.94(4.70-12.39) | 0.38(0.23-0.60) | 16.10(9.28-27.38) | 0.31(0.18-0.52) | -1.17(-1.46,-0.88) |
| **Cabo Verde** | 0.76(0.48-1.12) | 0.58(0.37-0.86) | 1.60(0.98-2.55) | 0.64(0.39-1.02) | 0.30(0.07,0.52) |
| **Cambodia** | 99.11(63.30-140.76) | 2.57(1.64-3.66) | 172.99(106.01-262.10) | 2.39(1.46-3.62) | -0.48(-0.63,-0.34) |
| **Cameroon** | 65.61(43.23-97.86) | 1.73(1.14-2.57) | 236.44(135.41-360.12) | 1.83(1.05-2.79) | -0.94(-1.59,-0.29) |
| **Canada** | 6.35(4.29-8.73) | 0.06(0.04-0.08) | 16.51(11.12-23.14) | 0.14(0.09-0.20) | 2.83(2.47,3.19) |
| **Central African Republic** | 7.91(4.84-11.55) | 0.76(0.46-1.11) | 18.05(10.13-30.13) | 0.83(0.46-1.38) | 0.19(0.09,0.30) |
| **Chad** | 13.54(8.25-21.88) | 0.64(0.39-1.04) | 46.14(27.42-76.94) | 0.73(0.44-1.22) | 0.42(0.21,0.62) |
| **Chile** | 10.18(6.89-14.48) | 0.18(0.12-0.25) | 8.89(5.78-12.68) | 0.13(0.08-0.18) | -1.24(-1.40,-1.09) |
| **China** | 3042.43(2106.07-4292.80) | 0.56(0.38-0.78) | 1556.85(982.90-2326.92) | 0.34(0.21-0.50) | -2.42(-2.78,-2.06) |
| **Colombia** | 32.53(22.22-46.05) | 0.23(0.16-0.33) | 23.08(15.00-34.01) | 0.11(0.07-0.17) | -1.94(-2.24,-1.63) |
| **Comoros** | 0.60(0.25-0.97) | 0.34(0.14-0.56) | 1.22(0.75-1.88) | 0.39(0.24-0.61) | -0.22(-0.84,0.40) |
| **Congo** | 8.18(4.90-12.36) | 0.86(0.52-1.30) | 20.47(11.40-33.54) | 0.92(0.51-1.51) | 0.14(-0.08,0.36) |
| **Cook Islands** | 0.01(0.01-0.02) | 0.16(0.09-0.26) | 0.01(0.01-0.02) | 0.21(0.11-0.35) | 1.23(0.97,1.48) |
| **Costa Rica** | 1.54(1.02-2.25) | 0.12(0.08-0.18) | 4.63(3.03-6.79) | 0.24(0.16-0.36) | 2.51(2.21,2.80) |
| **Côte d'Ivoire** | 45.95(28.57-69.74) | 0.97(0.60-1.47) | 126.47(74.50-197.97) | 1.13(0.66-1.77) | 0.48(0.33,0.63) |
| **Croatia** | 1.36(0.85-2.05) | 0.07(0.05-0.11) | 0.52(0.30-0.87) | 0.04(0.02-0.07) | -0.14(-0.81,0.53) |
| **Cuba** | 13.79(9.92-18.97) | 0.28(0.20-0.39) | 12.83(8.72-18.02) | 0.36(0.24-0.50) | 0.36(-0.01,0.72) |
| **Cyprus** | 0.10(0.06-0.16) | 0.03(0.02-0.05) | 0.11(0.07-0.18) | 0.02(0.01-0.04) | -1.86(-2.32,-1.39) |
| **Czechia** | 2.62(1.67-3.93) | 0.07(0.04-0.11) | 0.97(0.58-1.56) | 0.03(0.02-0.05) | -1.49(-1.90,-1.08) |
| **Democratic People's Republic of Korea** | 50.58(29.75-80.43) | 0.61(0.36-0.96) | 62.37(37.50-98.94) | 0.62(0.37-0.98) | -0.26(-0.38,-0.13) |
| **Democratic Republic of the Congo** | 84.87(51.29-127.42) | 0.59(0.36-0.89) | 225.83(131.97-357.91) | 0.63(0.37-0.99) | 0.06(-0.09,0.20) |
| **Denmark** | 0.26(0.16-0.40) | 0.01(0.01-0.02) | 0.17(0.13-0.23) | 0.01(0.01-0.01) | -1.32(-1.66,-0.98) |
| **Djibouti** | 0.38(0.22-0.61) | 0.22(0.12-0.35) | 1.83(1.03-2.99) | 0.34(0.19-0.55) | 1.36(1.03,1.68) |
| **Dominica** | 0.15(0.11-0.22) | 0.53(0.37-0.75) | 0.31(0.19-0.45) | 1.19(0.74-1.72) | 3.01(2.82,3.20) |
| **Dominican Republic** | 17.52(12.08-24.56) | 0.57(0.39-0.80) | 43.00(24.52-63.99) | 0.95(0.54-1.41) | 2.56(2.21,2.90) |
| **Ecuador** | 17.39(12.07-24.68) | 0.42(0.29-0.60) | 29.78(18.31-45.51) | 0.41(0.25-0.62) | -0.86(-1.89,0.18) |
| **Egypt** | 116.57(71.04-192.65) | 0.53(0.32-0.88) | 280.17(169.67-422.58) | 0.66(0.40-1.00) | 0.99(0.57,1.42) |
| **El Salvador** | 6.73(4.33-10.28) | 0.32(0.21-0.49) | 22.37(13.12-35.01) | 0.86(0.51-1.35) | 3.55(3.09,4.01) |
| **Equatorial Guinea** | 1.05(0.64-1.62) | 0.70(0.42-1.07) | 5.55(2.73-9.86) | 0.80(0.39-1.42) | 0.64(0.22,1.06) |
| **Eritrea** | 4.22(2.34-7.09) | 0.33(0.18-0.55) | 10.29(5.34-18.22) | 0.37(0.19-0.65) | 0.32(0.21,0.44) |
| **Estonia** | 1.11(0.72-1.64) | 0.20(0.13-0.29) | 0.71(0.52-0.97) | 0.18(0.13-0.25) | -1.17(-1.91,-0.43) |
| **Eswatini** | 1.46(0.87-2.27) | 0.48(0.29-0.75) | 5.50(2.68-8.91) | 1.08(0.53-1.75) | 2.90(2.11,3.70) |
| **Ethiopia** | 173.74(110.13-250.17) | 0.95(0.60-1.37) | 234.61(155.74-334.47) | 0.51(0.34-0.72) | -2.55(-2.75,-2.35) |
| **Fiji** | 1.18(0.68-1.96) | 0.37(0.21-0.61) | 2.02(1.14-3.36) | 0.57(0.32-0.94) | 1.08(0.90,1.25) |
| **Finland** | 0.07(0.04-0.11) | 0.00(0.00-0.01) | 0.05(0.04-0.07) | 0.00(0.00-0.00) | 0.08(-0.24,0.40) |
| **France** | 5.30(3.34-7.73) | 0.02(0.02-0.04) | 4.18(3.20-5.28) | 0.02(0.02-0.03) | 0.08(-0.15,0.32) |
| **Gabon** | 2.89(1.81-4.32) | 0.75(0.47-1.12) | 7.26(3.34-12.41) | 0.97(0.45-1.66) | 0.69(0.45,0.93) |
| **Gambia** | 3.23(1.89-4.82) | 0.86(0.50-1.28) | 11.76(7.41-18.10) | 1.18(0.74-1.81) | 0.66(0.37,0.96) |
| **Georgia** | 0.73(0.42-1.18) | 0.03(0.02-0.06) | 0.73(0.41-1.25) | 0.06(0.04-0.11) | 2.10(1.47,2.73) |
| **Germany** | 7.76(4.87-11.85) | 0.03(0.02-0.04) | 3.89(2.41-6.02) | 0.02(0.01-0.02) | -1.08(-1.43,-0.74) |
| **Ghana** | 33.60(19.62-52.84) | 0.59(0.34-0.92) | 128.20(78.75-195.82) | 0.90(0.55-1.37) | 1.59(1.42,1.75) |
| **Global** | 11293.77(8368.72-14947.73) | 0.52(0.38-0.68) | 16306.00(11671.08-21874.25) | 0.55(0.39-0.74) | -0.02(-0.13,0.10) |
| **Greece** | 1.00(0.65-1.50) | 0.03(0.02-0.04) | 0.99(0.73-1.33) | 0.04(0.03-0.05) | 2.34(1.96,2.73) |
| **Greenland** | 0.03(0.02-0.06) | 0.12(0.07-0.21) | 0.03(0.01-0.04) | 0.12(0.07-0.20) | -0.28(-0.51,-0.05) |
| **Grenada** | 0.40(0.28-0.55) | 1.19(0.84-1.64) | 0.61(0.41-0.86) | 1.51(1.01-2.13) | 1.27(0.93,1.61) |
| **Guam** | 0.35(0.23-0.50) | 0.55(0.37-0.79) | 0.63(0.42-0.90) | 1.14(0.75-1.62) | 3.01(2.75,3.26) |
| **Guatemala** | 10.37(6.91-14.86) | 0.35(0.23-0.50) | 36.31(23.45-53.25) | 0.53(0.34-0.78) | 1.73(1.34,2.13) |
| **Guinea** | 17.29(10.97-26.72) | 0.84(0.53-1.30) | 47.13(27.72-74.52) | 0.91(0.54-1.44) | 0.26(0.21,0.31) |
| **Guinea-Bissau** | 5.69(3.69-8.17) | 1.53(0.99-2.20) | 12.43(7.48-18.79) | 1.47(0.89-2.23) | -0.19(-0.27,-0.10) |
| **Guyana** | 2.78(1.94-3.91) | 0.82(0.57-1.15) | 4.58(2.82-6.72) | 1.47(0.91-2.16) | 2.49(1.97,3.00) |
| **Haiti** | 19.28(11.68-33.61) | 0.79(0.48-1.38) | 44.02(21.23-96.68) | 0.80(0.39-1.76) | 0.39(0.12,0.66) |
| **Honduras** | 2.49(1.57-3.75) | 0.14(0.09-0.22) | 5.59(2.64-9.57) | 0.13(0.06-0.22) | -0.95(-1.34,-0.55) |
| **Hungary** | 3.54(2.26-5.32) | 0.10(0.06-0.14) | 1.11(0.66-1.74) | 0.04(0.02-0.06) | -2.61(-3.08,-2.13) |
| **Iceland** | 0.01(0.01-0.02) | 0.01(0.01-0.02) | 0.02(0.02-0.03) | 0.02(0.01-0.02) | 1.76(1.32,2.21) |
| **India** | 889.76(566.92-1329.51) | 0.26(0.17-0.39) | 1692.02(1060.98-2471.33) | 0.28(0.17-0.41) | 0.20(0.03,0.36) |
| **Indonesia** | 1749.47(1146.89-2240.12) | 2.24(1.47-2.87) | 2843.08(1860.52-4008.49) | 2.50(1.63-3.52) | 0.47(0.35,0.59) |
| **Iran (Islamic Republic of)** | 63.13(42.79-95.27) | 0.29(0.20-0.44) | 152.63(102.38-210.43) | 0.44(0.30-0.61) | 1.55(1.40,1.70) |
| **Iraq** | 33.23(20.04-53.25) | 0.46(0.28-0.74) | 60.36(29.87-104.32) | 0.35(0.17-0.60) | -0.79(-1.04,-0.53) |
| **Ireland** | 0.17(0.11-0.25) | 0.01(0.01-0.02) | 0.20(0.13-0.31) | 0.01(0.01-0.02) | 0.82(0.40,1.23) |
| **Israel** | 0.94(0.62-1.39) | 0.05(0.03-0.07) | 1.59(0.98-2.40) | 0.05(0.03-0.07) | 0.41(0.12,0.70) |
| **Italy** | 6.66(4.43-9.59) | 0.03(0.02-0.04) | 3.45(2.12-5.85) | 0.02(0.01-0.04) | -1.83(-2.08,-1.58) |
| **Jamaica** | 3.91(2.78-5.22) | 0.40(0.28-0.53) | 10.16(5.91-15.39) | 0.85(0.50-1.29) | 1.28(0.49,2.08) |
| **Japan** | 16.76(10.82-24.86) | 0.04(0.02-0.06) | 5.95(3.81-8.88) | 0.02(0.01-0.03) | -1.76(-2.10,-1.42) |
| **Jordan** | 4.06(2.59-6.22) | 0.26(0.17-0.40) | 15.37(9.55-23.13) | 0.29(0.18-0.43) | 0.02(-0.28,0.32) |
| **Kazakhstan** | 3.15(1.95-4.84) | 0.05(0.03-0.07) | 3.04(1.70-4.88) | 0.04(0.02-0.07) | -1.64(-2.50,-0.77) |
| **Kenya** | 16.29(9.75-26.58) | 0.19(0.11-0.30) | 60.31(38.57-95.12) | 0.28(0.18-0.44) | 1.63(1.41,1.84) |
| **Kiribati** | 0.13(0.08-0.19) | 0.41(0.25-0.63) | 0.30(0.14-0.55) | 0.61(0.29-1.10) | 0.90(0.64,1.16) |
| **Kuwait** | 3.22(2.10-4.63) | 0.38(0.25-0.55) | 4.14(2.64-6.21) | 0.19(0.12-0.29) | -2.34(-2.90,-1.78) |
| **Kyrgyzstan** | 1.27(0.76-1.98) | 0.07(0.04-0.11) | 1.87(1.09-2.94) | 0.07(0.04-0.11) | -1.37(-2.08,-0.67) |
| **Lao People's Democratic Republic** | 51.40(32.67-72.12) | 3.33(2.11-4.67) | 93.06(58.51-141.09) | 2.90(1.82-4.40) | -0.63(-0.72,-0.53) |
| **Latvia** | 0.36(0.23-0.54) | 0.04(0.02-0.06) | 0.25(0.14-0.42) | 0.05(0.03-0.08) | -0.30(-1.13,0.53) |
| **Lebanon** | 4.69(2.71-7.71) | 0.41(0.24-0.67) | 7.64(4.71-11.22) | 0.33(0.20-0.48) | -0.82(-1.15,-0.48) |
| **Lesotho** | 1.36(0.80-2.24) | 0.25(0.15-0.42) | 6.42(3.67-10.24) | 0.77(0.44-1.23) | 4.83(4.31,5.36) |
| **Liberia** | 11.72(6.64-22.07) | 1.27(0.72-2.39) | 30.78(19.35-45.42) | 1.37(0.86-2.02) | 0.92(0.36,1.49) |
| **Libya** | 6.13(3.58-9.68) | 0.37(0.21-0.58) | 21.65(9.99-35.29) | 0.72(0.33-1.18) | 3.04(2.64,3.44) |
| **Lithuania** | 0.71(0.46-1.06) | 0.05(0.03-0.08) | 0.51(0.31-0.81) | 0.06(0.04-0.10) | -0.34(-0.77,0.08) |
| **Luxembourg** | 0.04(0.02-0.05) | 0.02(0.02-0.04) | 0.03(0.02-0.04) | 0.01(0.01-0.02) | -2.17(-2.44,-1.91) |
| **Madagascar** | 11.88(7.13-18.01) | 0.26(0.16-0.40) | 30.72(18.14-47.62) | 0.26(0.15-0.41) | 0.02(-0.06,0.10) |
| **Malawi** | 13.07(8.27-19.50) | 0.35(0.22-0.52) | 35.38(21.44-53.85) | 0.43(0.26-0.66) | 0.60(0.32,0.88) |
| **Malaysia** | 92.56(67.52-121.33) | 1.25(0.91-1.63) | 166.95(120.44-223.19) | 1.20(0.87-1.61) | -0.48(-0.75,-0.20) |
| **Maldives** | 2.15(1.44-2.89) | 2.64(1.77-3.55) | 4.24(2.85-6.01) | 1.63(1.09-2.31) | -1.55(-1.89,-1.21) |
| **Mali** | 28.73(18.65-43.89) | 0.96(0.62-1.47) | 74.31(45.16-116.30) | 0.83(0.51-1.31) | -0.40(-0.57,-0.23) |
| **Malta** | 0.03(0.02-0.04) | 0.02(0.01-0.03) | 0.03(0.02-0.05) | 0.02(0.02-0.04) | 1.44(1.15,1.72) |
| **Marshall Islands** | 0.08(0.04-0.15) | 0.47(0.23-0.86) | 0.20(0.05-0.57) | 0.86(0.23-2.39) | 2.10(1.94,2.26) |
| **Mauritania** | 7.37(4.79-10.79) | 0.96(0.62-1.40) | 12.75(7.60-20.65) | 0.75(0.45-1.21) | -1.01(-1.12,-0.91) |
| **Mauritius** | 11.73(8.94-14.90) | 2.36(1.80-3.00) | 22.41(17.20-29.28) | 4.92(3.78-6.43) | 2.20(1.92,2.48) |
| **Mexico** | 102.45(71.31-144.60) | 0.29(0.20-0.41) | 313.33(206.45-467.37) | 0.61(0.40-0.91) | 2.90(2.64,3.15) |
| **Micronesia (Federated States of)** | 0.18(0.10-0.30) | 0.45(0.24-0.75) | 0.32(0.17-0.53) | 0.75(0.40-1.24) | 1.69(1.38,2.00) |
| **Monaco** | 0.00(0.00-0.00) | 0.02(0.01-0.03) | 0.00(0.00-0.00) | 0.03(0.01-0.05) | 1.11(0.59,1.63) |
| **Mongolia** | 0.52(0.29-0.85) | 0.06(0.03-0.10) | 0.91(0.52-1.47) | 0.07(0.04-0.12) | 0.22(-0.16,0.60) |
| **Montenegro** | 0.62(0.39-0.96) | 0.25(0.15-0.38) | 0.46(0.28-0.68) | 0.22(0.14-0.33) | -0.19(-0.54,0.16) |
| **Morocco** | 18.31(10.87-31.25) | 0.18(0.10-0.30) | 31.04(17.56-56.01) | 0.21(0.12-0.38) | 0.79(0.62,0.96) |
| **Mozambique** | 11.45(6.78-18.68) | 0.24(0.14-0.39) | 48.19(27.21-76.56) | 0.40(0.23-0.64) | 2.40(2.14,2.66) |
| **Myanmar** | 621.91(387.65-892.47) | 3.62(2.26-5.20) | 578.76(387.81-821.79) | 2.57(1.72-3.66) | -1.44(-1.63,-1.25) |
| **Namibia** | 1.48(0.84-2.96) | 0.26(0.15-0.53) | 3.95(1.93-7.15) | 0.38(0.18-0.68) | 0.75(0.22,1.27) |
| **Nauru** | 0.02(0.01-0.04) | 0.55(0.29-0.91) | 0.04(0.02-0.06) | 0.86(0.48-1.38) | 1.22(1.08,1.35) |
| **Nepal** | 20.38(12.42-32.26) | 0.28(0.17-0.44) | 44.02(26.03-70.12) | 0.33(0.19-0.52) | 0.67(0.36,0.98) |
| **Netherlands** | 1.14(0.76-1.68) | 0.02(0.01-0.03) | 1.18(0.91-1.50) | 0.02(0.02-0.03) | 1.53(1.16,1.90) |
| **New Zealand** | 0.15(0.10-0.22) | 0.01(0.01-0.02) | 0.30(0.19-0.44) | 0.02(0.01-0.02) | 1.67(1.37,1.98) |
| **Nicaragua** | 4.68(3.08-7.08) | 0.32(0.21-0.48) | 21.27(12.46-32.90) | 0.75(0.44-1.16) | 3.39(3.08,3.70) |
| **Niger** | 19.77(12.08-33.13) | 0.71(0.43-1.19) | 50.86(28.41-94.98) | 0.57(0.32-1.07) | -0.93(-1.11,-0.76) |
| **Nigeria** | 180.00(105.00-277.27) | 0.53(0.31-0.81) | 519.71(284.80-901.60) | 0.58(0.32-1.00) | 0.39(0.16,0.61) |
| **Niue** | 0.00(0.00-0.00) | 0.35(0.19-0.57) | 0.00(0.00-0.01) | 0.79(0.36-1.41) | 1.74(1.45,2.04) |
| **North Macedonia** | 0.78(0.48-1.19) | 0.10(0.06-0.15) | 0.63(0.36-1.11) | 0.08(0.05-0.15) | -0.34(-0.55,-0.13) |
| **Northern Mariana Islands** | 0.04(0.02-0.07) | 0.17(0.08-0.29) | 0.04(0.02-0.06) | 0.22(0.12-0.37) | 0.59(0.39,0.79) |
| **Norway** | 0.16(0.10-0.23) | 0.01(0.01-0.01) | 0.23(0.15-0.34) | 0.01(0.01-0.02) | 1.98(1.44,2.52) |
| **Oman** | 2.08(1.12-3.71) | 0.25(0.13-0.45) | 8.42(4.53-13.12) | 0.36(0.20-0.57) | 2.07(1.67,2.46) |
| **Pakistan** | 135.78(87.08-207.18) | 0.33(0.21-0.51) | 546.17(335.25-856.41) | 0.55(0.34-0.87) | 1.35(1.08,1.63) |
| **Palau** | 0.03(0.01-0.05) | 0.39(0.20-0.66) | 0.05(0.03-0.09) | 0.89(0.43-1.54) | 2.83(2.63,3.03) |
| **Palestine** | 2.67(1.52-4.42) | 0.35(0.20-0.58) | 7.52(4.76-11.24) | 0.34(0.22-0.51) | -0.29(-0.54,-0.03) |
| **Panama** | 1.31(0.90-1.96) | 0.13(0.09-0.19) | 4.44(2.75-6.67) | 0.27(0.17-0.40) | 2.51(1.89,3.14) |
| **Papua New Guinea** | 3.76(1.45-6.49) | 0.23(0.09-0.39) | 12.19(7.11-19.24) | 0.28(0.17-0.45) | 0.54(0.40,0.69) |
| **Paraguay** | 3.53(2.34-5.29) | 0.22(0.15-0.34) | 9.04(5.44-14.09) | 0.30(0.18-0.46) | 0.87(0.72,1.02) |
| **Peru** | 37.36(24.82-54.22) | 0.42(0.28-0.61) | 57.60(33.67-90.37) | 0.39(0.23-0.61) | -0.44(-0.65,-0.22) |
| **Philippines** | 661.96(503.16-841.65) | 2.55(1.94-3.25) | 1531.58(1100.35-1983.87) | 3.24(2.33-4.20) | 1.25(1.01,1.49) |
| **Poland** | 17.53(10.96-26.63) | 0.12(0.08-0.18) | 3.63(2.20-5.73) | 0.03(0.02-0.05) | -2.42(-3.25,-1.58) |
| **Portugal** | 1.60(1.06-2.39) | 0.04(0.03-0.06) | 0.83(0.50-1.26) | 0.03(0.02-0.04) | -1.81(-2.18,-1.44) |
| **Puerto Rico** | 6.62(4.41-9.75) | 0.47(0.31-0.69) | 5.67(4.41-7.07) | 0.55(0.43-0.68) | 0.87(0.46,1.28) |
| **Qatar** | 0.67(0.31-1.22) | 0.28(0.13-0.52) | 4.50(2.72-6.77) | 0.27(0.16-0.41) | -0.74(-1.22,-0.25) |
| **Republic of Korea** | 43.16(30.87-53.55) | 0.21(0.15-0.25) | 9.98(6.23-14.90) | 0.06(0.04-0.09) | -3.75(-4.09,-3.41) |
| **Republic of Moldova** | 0.61(0.39-0.90) | 0.03(0.02-0.05) | 0.54(0.33-0.86) | 0.04(0.03-0.07) | -0.49(-1.00,0.03) |
| **Romania** | 4.48(3.37-5.82) | 0.05(0.04-0.07) | 1.42(0.93-2.10) | 0.03(0.02-0.04) | -1.99(-2.47,-1.50) |
| **Russian Federation** | 75.99(49.27-111.41) | 0.13(0.08-0.19) | 24.22(15.23-37.29) | 0.05(0.03-0.08) | -4.43(-4.94,-3.93) |
| **Rwanda** | 14.60(9.28-22.18) | 0.53(0.34-0.81) | 18.91(11.41-29.12) | 0.33(0.20-0.51) | -2.52(-3.03,-2.01) |
| **Saint Kitts and Nevis** | 0.19(0.13-0.27) | 1.12(0.77-1.56) | 0.21(0.13-0.36) | 0.94(0.58-1.58) | -1.10(-1.92,-0.27) |
| **Saint Lucia** | 0.46(0.33-0.62) | 0.81(0.59-1.11) | 0.86(0.56-1.19) | 1.30(0.84-1.80) | 1.95(1.74,2.17) |
| **Saint Vincent and the Grenadines** | 0.32(0.23-0.44) | 0.70(0.50-0.95) | 0.48(0.32-0.67) | 1.16(0.77-1.62) | 2.02(1.69,2.35) |
| **Samoa** | 0.22(0.12-0.37) | 0.33(0.18-0.55) | 0.44(0.24-0.73) | 0.55(0.30-0.91) | 1.57(1.45,1.70) |
| **San Marino** | 0.00(0.00-0.00) | 0.01(0.01-0.01) | 0.00(0.00-0.00) | 0.01(0.00-0.01) | 0.62(0.08,1.16) |
| **Sao Tome and Principe** | 0.46(0.25-0.70) | 1.07(0.58-1.62) | 1.35(0.74-2.27) | 1.49(0.82-2.50) | 0.85(0.40,1.30) |
| **Saudi Arabia** | 32.40(19.38-52.27) | 0.49(0.29-0.79) | 238.69(126.81-388.82) | 1.29(0.68-2.10) | 3.42(3.24,3.59) |
| **Senegal** | 28.26(18.26-42.05) | 1.02(0.66-1.52) | 65.98(38.42-110.22) | 1.02(0.60-1.71) | 0.25(0.05,0.45) |
| **Serbia** | 8.65(5.62-13.19) | 0.24(0.16-0.37) | 4.68(3.24-6.53) | 0.16(0.11-0.22) | -1.35(-1.50,-1.21) |
| **Seychelles** | 0.61(0.46-0.78) | 1.94(1.47-2.51) | 1.03(0.73-1.41) | 2.69(1.90-3.67) | 1.42(1.15,1.69) |
| **Sierra Leone** | 11.17(6.00-18.63) | 0.70(0.38-1.17) | 28.35(17.04-47.74) | 0.76(0.46-1.28) | 0.29(0.15,0.42) |
| **Singapore** | 0.75(0.49-1.13) | 0.05(0.03-0.07) | 0.39(0.25-0.60) | 0.02(0.01-0.03) | -1.00(-1.65,-0.34) |
| **Slovakia** | 1.99(1.22-3.07) | 0.10(0.06-0.15) | 1.12(0.64-1.78) | 0.07(0.04-0.10) | -0.49(-1.13,0.14) |
| **Slovenia** | 0.19(0.14-0.26) | 0.02(0.02-0.03) | 0.09(0.05-0.15) | 0.02(0.01-0.03) | -0.73(-1.19,-0.26) |
| **Solomon Islands** | 0.51(0.12-0.98) | 0.40(0.09-0.77) | 1.45(0.77-2.28) | 0.53(0.28-0.83) | 0.93(0.67,1.18) |
| **Somalia** | 12.41(7.13-21.98) | 0.43(0.25-0.76) | 35.49(19.07-62.57) | 0.43(0.23-0.75) | 0.29(0.14,0.45) |
| **South Africa** | 129.25(86.73-178.30) | 0.82(0.55-1.13) | 247.43(158.96-347.37) | 1.02(0.66-1.43) | -0.52(-1.40,0.37) |
| **South Sudan** | 6.32(3.78-9.91) | 0.27(0.16-0.43) | 15.98(9.36-25.48) | 0.44(0.26-0.71) | 1.52(1.15,1.90) |
| **Spain** | 4.55(2.96-6.78) | 0.03(0.02-0.05) | 2.08(1.28-3.20) | 0.02(0.01-0.03) | -2.20(-2.40,-2.00) |
| **Sri Lanka** | 141.82(106.94-190.77) | 1.92(1.45-2.58) | 114.96(72.12-168.58) | 1.43(0.89-2.09) | -1.46(-1.79,-1.13) |
| **Sudan** | 36.26(21.03-61.60) | 0.48(0.28-0.81) | 108.75(56.95-169.73) | 0.59(0.31-0.92) | 0.88(0.77,1.00) |
| **Suriname** | 1.37(0.89-1.95) | 0.84(0.55-1.19) | 2.93(1.87-4.34) | 1.37(0.87-2.02) | 1.62(1.37,1.88) |
| **Sweden** | 0.19(0.12-0.29) | 0.01(0.00-0.01) | 0.33(0.20-0.53) | 0.01(0.01-0.02) | 1.82(1.47,2.17) |
| **Switzerland** | 0.86(0.58-1.21) | 0.03(0.02-0.05) | 0.53(0.39-0.71) | 0.02(0.01-0.03) | -1.81(-2.07,-1.55) |
| **Syrian Arab Republic** | 28.14(17.43-41.73) | 0.59(0.36-0.87) | 25.05(13.84-46.06) | 0.49(0.27-0.91) | -0.35(-0.97,0.27) |
| **Taiwan (Province of China)** | 41.08(29.49-56.12) | 0.45(0.32-0.61) | 18.82(13.18-27.15) | 0.25(0.17-0.36) | -1.37(-1.67,-1.07) |
| **Tajikistan** | 0.29(0.17-0.48) | 0.01(0.01-0.02) | 0.75(0.40-1.38) | 0.02(0.01-0.03) | -0.59(-1.16,-0.02) |
| **Thailand** | 465.40(333.45-674.47) | 1.79(1.29-2.60) | 467.71(306.07-685.77) | 2.21(1.44-3.23) | -1.01(-1.86,-0.15) |
| **Timor-Leste** | 5.65(3.43-8.42) | 1.77(1.08-2.65) | 9.92(5.97-15.67) | 1.74(1.04-2.74) | -0.10(-0.79,0.58) |
| **Togo** | 10.80(6.79-15.88) | 0.79(0.50-1.16) | 31.10(18.85-49.35) | 0.92(0.56-1.47) | 0.52(0.40,0.64) |
| **Tokelau** | 0.00(0.00-0.00) | 0.29(0.16-0.54) | 0.00(0.00-0.01) | 0.64(0.37-1.01) | 1.63(1.23,2.04) |
| **Tonga** | 0.04(0.02-0.07) | 0.11(0.06-0.19) | 0.07(0.04-0.12) | 0.18(0.10-0.30) | 1.54(1.37,1.71) |
| **Trinidad and Tobago** | 3.41(2.42-4.70) | 0.68(0.48-0.94) | 6.93(4.07-10.49) | 1.39(0.82-2.11) | 2.37(2.12,2.63) |
| **Tunisia** | 4.63(2.86-7.72) | 0.13(0.08-0.22) | 9.74(5.22-16.00) | 0.22(0.12-0.37) | 1.61(1.44,1.78) |
| **Turkey** | 89.92(58.26-135.46) | 0.38(0.24-0.57) | 77.85(47.72-116.01) | 0.24(0.15-0.36) | -0.94(-1.42,-0.45) |
| **Turkmenistan** | 1.01(0.62-1.54) | 0.07(0.04-0.10) | 2.72(1.59-4.25) | 0.13(0.08-0.20) | 2.11(1.72,2.49) |
| **Tuvalu** | 0.01(0.01-0.02) | 0.40(0.23-0.66) | 0.03(0.01-0.04) | 0.54(0.29-0.88) | 0.92(0.85,1.00) |
| **Uganda** | 12.93(7.33-20.55) | 0.20(0.11-0.32) | 53.09(31.40-84.35) | 0.31(0.18-0.49) | 0.71(0.37,1.05) |
| **Ukraine** | 0.67(0.42-0.97) | 0.00(0.00-0.01) | 10.13(5.68-16.06) | 0.07(0.04-0.12) | 12.35(10.54,14.20) |
| **United Arab Emirates** | 2.08(1.12-3.72) | 0.22(0.12-0.39) | 10.52(3.40-18.63) | 0.26(0.08-0.46) | 0.71(0.40,1.02) |
| **United Kingdom** | 2.10(1.37-3.13) | 0.01(0.01-0.01) | 2.91(1.86-4.42) | 0.01(0.01-0.02) | 1.32(1.03,1.61) |
| **United Republic of Tanzania** | 15.94(9.93-24.90) | 0.16(0.10-0.26) | 40.94(24.41-65.25) | 0.18(0.10-0.28) | -0.03(-0.14,0.08) |
| **United States of America** | 114.02(73.40-167.32) | 0.11(0.07-0.16) | 348.97(280.69-419.40) | 0.31(0.25-0.38) | 3.96(3.62,4.31) |
| **United States Virgin Islands** | 0.37(0.25-0.51) | 0.93(0.64-1.29) | 0.46(0.29-0.67) | 2.00(1.27-2.90) | 3.18(2.97,3.39) |
| **Uruguay** | 1.38(1.23-1.56) | 0.12(0.11-0.14) | 1.84(1.24-2.60) | 0.15(0.10-0.22) | 1.14(0.81,1.48) |
| **Uzbekistan** | 2.00(1.16-3.23) | 0.02(0.01-0.04) | 11.38(6.92-17.45) | 0.08(0.05-0.13) | 2.57(1.73,3.42) |
| **Vanuatu** | 0.16(0.07-0.30) | 0.27(0.12-0.51) | 0.58(0.31-0.99) | 0.46(0.25-0.80) | 1.61(1.51,1.72) |
| **Venezuela (Bolivarian Republic of)** | 14.89(10.09-21.74) | 0.19(0.13-0.27) | 39.72(23.49-62.60) | 0.42(0.25-0.67) | 2.26(1.90,2.62) |
| **Viet Nam** | 489.64(270.46-691.49) | 1.72(0.95-2.42) | 601.20(354.79-881.10) | 1.57(0.92-2.30) | -0.27(-0.60,0.06) |
| **Yemen** | 9.44(4.18-18.48) | 0.21(0.09-0.40) | 26.52(13.04-52.49) | 0.19(0.09-0.38) | -0.02(-0.20,0.15) |
| **Zambia** | 12.55(7.94-19.47) | 0.41(0.26-0.64) | 42.97(22.89-80.62) | 0.53(0.28-1.00) | 0.59(0.45,0.73) |
| **Zimbabwe** | 10.68(6.17-20.26) | 0.27(0.16-0.51) | 46.93(26.06-82.24) | 0.74(0.41-1.30) | 4.15(3.30,5.01) |
| ***ASDR Age-standardized death rate, EAPC Estimated annual percentage change, CI Confidence interval, UI Uncertainty interval*** | | | | | |

| **eTable 3 Cases and ASR of DALYs to CKD due to hypertension in 1990 and 2021, and temporal trends in 204 Countries and Territories** | | | | | |
| --- | --- | --- | --- | --- | --- |
|  | **1990** |  | **2021** |  | **1990-2021** |
|  | **DALYs cases (95% UI)** | **ASR of DALYs (per 100,000 population)** | **DALYs cases (95% UI)** | **ASR of DALYs (per 100,000 population)** | **EAPCs of DALYs ASR(95% CI)** |
| **Afghanistan** | 1377.53(879.00-2200.62) | 43.74(27.91-69.87) | 5315.88(2896.23-8694.54) | 43.50(23.70-71.14) | 0.31(-0.07,0.69) |
| **Albania** | 140.42(95.38-204.72) | 9.89(6.71-14.41) | 77.60(50.21-108.04) | 8.19(5.30-11.40) | -0.95(-1.13,-0.78) |
| **Algeria** | 2363.15(1604.40-3584.60) | 23.39(15.88-35.48) | 5153.96(3488.04-7296.52) | 30.27(20.49-42.85) | 0.83(0.61,1.04) |
| **American Samoa** | 5.16(3.38-7.47) | 25.49(16.69-36.90) | 9.50(6.11-14.63) | 54.35(34.99-83.73) | 2.55(2.30,2.80) |
| **Andorra** | 1.93(1.19-2.98) | 7.72(4.75-11.92) | 1.90(1.09-3.18) | 7.45(4.29-12.49) | -0.15(-0.25,-0.05) |
| **Angola** | 1668.77(1111.11-2444.22) | 42.65(28.40-62.47) | 5216.15(3356.99-8024.74) | 42.87(27.59-65.96) | 0.19(0.03,0.35) |
| **Antigua and Barbuda** | 13.04(9.82-17.29) | 50.55(38.09-67.04) | 19.03(14.33-26.14) | 55.32(41.66-76.00) | 1.44(0.94,1.93) |
| **Argentina** | 2925.76(2173.75-3913.52) | 23.95(17.80-32.04) | 3085.59(2331.22-4136.29) | 17.61(13.30-23.61) | -0.72(-0.83,-0.61) |
| **Armenia** | 115.31(66.46-187.52) | 8.02(4.62-13.05) | 107.64(66.06-165.74) | 10.01(6.14-15.42) | 0.47(0.34,0.60) |
| **Australia** | 203.76(117.60-337.65) | 3.01(1.74-4.99) | 300.68(174.09-490.41) | 3.47(2.01-5.66) | 0.09(-0.04,0.23) |
| **Austria** | 198.15(125.84-308.38) | 6.60(4.19-10.27) | 214.27(134.24-330.41) | 7.59(4.76-11.71) | 0.45(0.37,0.53) |
| **Azerbaijan** | 385.05(237.40-590.61) | 12.12(7.47-18.58) | 625.95(387.85-970.43) | 14.78(9.16-22.91) | 0.28(0.15,0.41) |
| **Bahamas** | 61.92(46.45-83.94) | 52.47(39.36-71.12) | 135.80(91.81-189.89) | 87.81(59.36-122.78) | 1.76(1.60,1.92) |
| **Bahrain** | 60.49(41.85-90.74) | 23.60(16.33-35.40) | 224.31(156.42-311.39) | 31.88(22.23-44.26) | 0.58(0.29,0.87) |
| **Bangladesh** | 9309.17(6626.13-12851.31) | 22.05(15.70-30.45) | 12863.30(8593.35-18484.60) | 18.69(12.49-26.86) | -0.44(-0.64,-0.25) |
| **Barbados** | 44.13(33.50-57.58) | 40.43(30.69-52.75) | 47.40(33.57-66.13) | 47.98(33.99-66.95) | 0.65(0.39,0.91) |
| **Belarus** | 200.81(123.08-331.22) | 5.09(3.12-8.40) | 184.31(114.83-290.11) | 6.28(3.91-9.89) | 0.36(0.25,0.48) |
| **Belgium** | 305.11(194.32-459.96) | 8.20(5.22-12.36) | 290.65(176.06-459.58) | 8.30(5.03-13.12) | -0.15(-0.27,-0.03) |
| **Belize** | 28.47(21.62-37.41) | 38.91(29.56-51.13) | 155.17(114.62-214.50) | 82.19(60.71-113.61) | 2.87(2.44,3.30) |
| **Benin** | 993.74(666.17-1426.29) | 58.36(39.12-83.76) | 3287.32(2161.70-4755.82) | 62.70(41.23-90.72) | 0.10(-0.03,0.24) |
| **Bermuda** | 6.81(5.16-8.92) | 26.48(20.08-34.67) | 4.67(3.40-6.31) | 26.69(19.39-36.04) | 0.26(0.02,0.50) |
| **Bhutan** | 65.10(42.24-95.01) | 24.16(15.68-35.26) | 104.15(67.46-153.69) | 30.05(19.46-44.34) | 0.45(0.37,0.53) |
| **Bolivia (Plurinational State of)** | 966.31(665.85-1432.14) | 39.18(27.00-58.07) | 1725.75(1086.40-2614.28) | 35.12(22.11-53.20) | -0.73(-0.91,-0.54) |
| **Bosnia and Herzegovina** | 144.82(100.48-205.69) | 7.63(5.29-10.83) | 78.85(52.97-114.78) | 7.84(5.26-11.41) | 0.04(-0.34,0.43) |
| **Botswana** | 136.90(82.21-240.11) | 26.60(15.97-46.65) | 367.26(225.27-662.57) | 34.47(21.14-62.19) | 0.84(0.61,1.06) |
| **Brazil** | 18199.61(13908.45-24256.32) | 29.01(22.17-38.66) | 17818.37(13391.19-23922.75) | 20.90(15.71-28.06) | -1.27(-1.47,-1.07) |
| **Brunei Darussalam** | 28.54(20.37-39.32) | 23.15(16.52-31.90) | 46.23(32.64-64.66) | 22.66(16.00-31.69) | -0.26(-0.48,-0.04) |
| **Bulgaria** | 325.63(225.51-455.49) | 10.94(7.58-15.30) | 304.67(208.69-433.02) | 16.04(10.99-22.80) | 1.75(1.47,2.03) |
| **Burkina Faso** | 1766.95(1188.43-2529.04) | 55.43(37.28-79.33) | 5577.97(3627.92-8163.81) | 64.45(41.92-94.33) | 0.65(0.47,0.82) |
| **Burundi** | 566.12(377.13-831.72) | 27.31(18.19-40.12) | 1156.91(735.77-1851.53) | 21.94(13.96-35.12) | -1.07(-1.33,-0.82) |
| **Cabo Verde** | 51.48(34.71-72.65) | 39.37(26.54-55.56) | 106.58(71.07-158.97) | 42.53(28.36-63.43) | 0.23(0.05,0.42) |
| **Cambodia** | 6494.60(4263.82-9141.77) | 168.68(110.74-237.43) | 11116.82(7087.54-16605.31) | 153.48(97.85-229.26) | -0.53(-0.65,-0.40) |
| **Cameroon** | 4148.66(2816.03-6019.46) | 109.08(74.04-158.27) | 15047.72(9219.42-22526.50) | 116.71(71.51-174.72) | -0.82(-1.43,-0.21) |
| **Canada** | 919.56(660.61-1304.65) | 8.27(5.94-11.74) | 1762.29(1300.43-2345.80) | 14.86(10.96-19.78) | 1.76(1.52,2.01) |
| **Central African Republic** | 535.14(347.02-775.36) | 51.39(33.32-74.46) | 1209.58(721.62-1910.01) | 55.40(33.05-87.49) | 0.17(0.08,0.26) |
| **Chad** | 907.82(577.70-1391.54) | 43.25(27.52-66.29) | 3103.84(1961.84-4894.23) | 49.32(31.18-77.77) | 0.42(0.23,0.60) |
| **Chile** | 905.52(672.46-1182.15) | 15.81(11.74-20.64) | 907.71(686.56-1202.08) | 12.83(9.70-16.99) | -0.65(-0.74,-0.57) |
| **China** | 214995.91(160140.10-289852.84) | 39.22(29.22-52.88) | 113707.61(80949.89-158673.19) | 24.64(17.54-34.39) | -2.17(-2.45,-1.89) |
| **Colombia** | 2847.30(2094.56-3862.22) | 20.26(14.90-27.48) | 2495.03(1717.86-3468.31) | 12.42(8.55-17.26) | -1.49(-1.66,-1.31) |
| **Comoros** | 43.87(22.96-67.03) | 25.40(13.29-38.80) | 85.46(57.09-123.94) | 27.59(18.43-40.01) | -0.31(-0.84,0.23) |
| **Congo** | 546.97(351.77-791.58) | 57.74(37.13-83.56) | 1346.33(825.61-2084.58) | 60.77(37.26-94.09) | 0.08(-0.11,0.28) |
| **Cook Islands** | 1.29(0.88-1.85) | 16.69(11.46-23.99) | 1.23(0.81-1.80) | 20.80(13.75-30.55) | 0.82(0.68,0.96) |
| **Costa Rica** | 190.34(133.36-260.34) | 14.82(10.38-20.27) | 423.00(306.58-579.58) | 22.22(16.11-30.45) | 1.36(1.21,1.50) |
| **Côte d'Ivoire** | 3012.90(1967.99-4430.12) | 63.70(41.61-93.66) | 8108.06(4899.80-12274.49) | 72.32(43.70-109.48) | 0.40(0.25,0.54) |
| **Croatia** | 155.87(111.02-214.71) | 8.59(6.12-11.83) | 82.79(55.15-119.21) | 6.63(4.42-9.55) | -0.00(-0.30,0.30) |
| **Cuba** | 1086.09(838.14-1415.81) | 22.26(17.18-29.01) | 957.23(707.66-1300.89) | 26.70(19.74-36.28) | 0.24(-0.03,0.52) |
| **Cyprus** | 26.20(16.16-39.56) | 8.52(5.25-12.86) | 41.40(23.59-67.05) | 8.25(4.70-13.36) | -0.43(-0.60,-0.26) |
| **Czechia** | 331.60(225.37-461.50) | 8.94(6.07-12.44) | 192.65(125.52-288.00) | 6.52(4.25-9.75) | -0.53(-0.76,-0.30) |
| **Democratic People's Republic of Korea** | 3688.62(2377.16-5543.38) | 44.22(28.50-66.46) | 4464.94(2954.28-6674.85) | 44.39(29.37-66.36) | -0.30(-0.42,-0.19) |
| **Democratic Republic of the Congo** | 6001.09(3954.11-8500.47) | 41.89(27.60-59.34) | 15708.08(10349.34-23625.10) | 43.53(28.68-65.47) | -0.00(-0.13,0.12) |
| **Denmark** | 126.22(75.00-194.97) | 6.62(3.93-10.22) | 129.09(68.26-206.75) | 7.08(3.74-11.34) | 0.03(-0.14,0.21) |
| **Djibouti** | 29.76(19.46-44.48) | 16.97(11.10-25.37) | 131.10(84.13-203.21) | 24.24(15.56-37.58) | 1.10(0.83,1.37) |
| **Dominica** | 11.98(8.89-15.74) | 41.03(30.43-53.90) | 20.97(14.37-29.50) | 80.96(55.50-113.92) | 2.47(2.32,2.62) |
| **Dominican Republic** | 1210.92(869.70-1621.87) | 39.42(28.31-52.80) | 2829.77(1746.46-4065.14) | 62.23(38.41-89.40) | 2.29(1.96,2.61) |
| **Ecuador** | 1237.63(916.98-1706.44) | 29.99(22.22-41.35) | 2140.26(1463.61-3171.54) | 29.30(20.04-43.42) | -0.70(-1.60,0.20) |
| **Egypt** | 8274.69(5754.87-12572.87) | 37.75(26.25-57.35) | 19419.85(13016.64-27373.34) | 46.01(30.84-64.85) | 0.88(0.57,1.19) |
| **El Salvador** | 498.46(350.43-685.76) | 23.86(16.77-32.82) | 1480.46(914.59-2202.42) | 57.12(35.29-84.97) | 3.22(2.80,3.65) |
| **Equatorial Guinea** | 72.10(46.81-104.86) | 47.75(31.00-69.44) | 373.50(204.17-634.52) | 53.69(29.35-91.22) | 0.54(0.19,0.89) |
| **Eritrea** | 309.22(195.15-494.79) | 23.86(15.06-38.18) | 729.24(423.86-1211.84) | 26.04(15.14-43.28) | 0.22(0.12,0.32) |
| **Estonia** | 88.81(64.43-123.35) | 15.64(11.34-21.72) | 64.00(48.17-81.51) | 16.18(12.18-20.61) | -0.47(-1.05,0.11) |
| **Eswatini** | 102.28(67.42-148.66) | 33.94(22.37-49.33) | 351.02(192.65-550.29) | 68.95(37.84-108.08) | 2.57(1.88,3.28) |
| **Ethiopia** | 11425.79(7629.48-16128.45) | 62.54(41.76-88.28) | 15687.79(10888.80-22089.05) | 33.84(23.49-47.65) | -2.48(-2.66,-2.29) |
| **Fiji** | 104.21(66.98-157.62) | 32.32(20.77-48.88) | 168.70(108.85-250.27) | 47.29(30.51-70.16) | 1.00(0.87,1.13) |
| **Finland** | 89.67(48.24-150.83) | 4.94(2.66-8.31) | 84.87(42.60-142.50) | 5.09(2.56-8.55) | 0.11(-0.04,0.25) |
| **France** | 1108.34(712.46-1746.08) | 5.04(3.24-7.94) | 955.68(628.06-1466.24) | 4.81(3.16-7.38) | -0.11(-0.23,0.01) |
| **Gabon** | 196.63(132.79-280.58) | 51.12(34.52-72.95) | 481.44(262.05-766.48) | 64.23(34.96-102.26) | 0.62(0.40,0.83) |
| **Gambia** | 210.63(129.98-310.32) | 55.87(34.47-82.31) | 755.77(483.16-1136.72) | 75.57(48.31-113.66) | 0.64(0.36,0.91) |
| **Georgia** | 217.97(139.64-331.04) | 10.24(6.56-15.55) | 145.40(94.48-221.97) | 12.81(8.33-19.56) | 0.66(0.51,0.80) |
| **Germany** | 2263.02(1431.20-3476.18) | 7.62(4.82-11.70) | 1840.11(1042.04-2919.60) | 7.27(4.12-11.54) | -0.35(-0.55,-0.15) |
| **Ghana** | 2192.01(1404.94-3321.13) | 38.19(24.48-57.86) | 8160.72(5077.43-12203.63) | 57.06(35.50-85.33) | 1.51(1.34,1.68) |
| **Global** | 825628.28(646823.70-1054246.62) | 37.67(29.51-48.10) | 1180452.47(889946.51-1523802.55) | 39.68(29.92-51.22) | -0.01(-0.10,0.08) |
| **Greece** | 294.27(196.17-463.49) | 7.83(5.22-12.33) | 243.94(159.82-382.67) | 8.76(5.74-13.75) | 0.83(0.72,0.94) |
| **Greenland** | 3.23(2.27-4.74) | 12.19(8.57-17.92) | 2.62(1.84-3.60) | 12.84(9.03-17.65) | -0.34(-0.58,-0.09) |
| **Grenada** | 26.01(18.84-34.81) | 78.01(56.50-104.42) | 39.14(27.68-53.49) | 96.83(68.49-132.35) | 1.19(0.88,1.50) |
| **Guam** | 26.12(19.13-36.44) | 41.19(30.16-57.44) | 43.15(30.81-58.70) | 77.84(55.57-105.89) | 2.62(2.40,2.83) |
| **Guatemala** | 822.89(606.36-1124.84) | 27.86(20.53-38.08) | 2729.47(1968.62-3759.36) | 40.10(28.92-55.23) | 1.49(1.17,1.80) |
| **Guinea** | 1134.25(765.51-1692.39) | 55.21(37.26-82.38) | 3112.82(1994.02-4717.30) | 60.24(38.59-91.30) | 0.30(0.25,0.34) |
| **Guinea-Bissau** | 362.10(247.37-508.14) | 97.64(66.70-137.03) | 789.46(497.47-1170.41) | 93.56(58.96-138.71) | -0.19(-0.26,-0.12) |
| **Guyana** | 193.63(143.83-260.41) | 56.89(42.26-76.51) | 294.75(194.13-416.68) | 94.86(62.47-134.09) | 2.12(1.70,2.55) |
| **Haiti** | 1306.18(852.75-2133.08) | 53.66(35.03-87.63) | 3007.31(1716.95-6136.36) | 54.78(31.28-111.79) | 0.41(0.17,0.65) |
| **Honduras** | 269.62(191.92-371.13) | 15.62(11.12-21.50) | 618.04(384.44-949.87) | 14.06(8.75-21.61) | -0.68(-0.88,-0.47) |
| **Hungary** | 380.46(266.92-529.48) | 10.29(7.22-14.33) | 191.08(127.39-277.92) | 6.94(4.63-10.10) | -0.96(-1.24,-0.68) |
| **Iceland** | 4.30(2.71-6.64) | 4.14(2.61-6.39) | 6.52(3.96-10.43) | 5.45(3.31-8.72) | 1.07(0.94,1.21) |
| **India** | 79368.85(59357.71-107928.43) | 23.27(17.41-31.65) | 155593.35(115663.14-210843.89) | 25.53(18.98-34.60) | 0.36(0.20,0.52) |
| **Indonesia** | 114726.87(77007.39-146445.85) | 147.00(98.67-187.65) | 181919.81(123869.67-253286.52) | 159.75(108.78-222.43) | 0.35(0.25,0.46) |
| **Iran (Islamic Republic of)** | 5140.33(3907.51-7088.81) | 23.67(17.99-32.64) | 11225.75(8299.10-14423.74) | 32.35(23.91-41.56) | 1.40(1.23,1.57) |
| **Iraq** | 2504.42(1668.92-3642.74) | 34.86(23.23-50.70) | 4803.13(2866.12-7324.18) | 27.55(16.44-42.00) | -0.69(-0.89,-0.50) |
| **Ireland** | 97.57(61.01-151.73) | 7.11(4.45-11.06) | 135.55(78.55-218.83) | 8.66(5.02-13.99) | 0.68(0.47,0.89) |
| **Israel** | 198.86(133.08-290.80) | 10.41(6.96-15.22) | 337.96(223.81-480.31) | 10.17(6.74-14.45) | 0.04(-0.05,0.12) |
| **Italy** | 1916.59(1331.97-2648.02) | 8.98(6.24-12.40) | 1460.65(979.73-2086.54) | 9.25(6.20-13.21) | -0.10(-0.24,0.03) |
| **Jamaica** | 298.35(226.52-387.14) | 30.36(23.05-39.39) | 678.22(439.25-973.65) | 56.84(36.81-81.60) | 1.02(0.36,1.69) |
| **Japan** | 2304.63(1698.99-3083.49) | 5.14(3.79-6.88) | 1418.49(992.86-2020.63) | 4.38(3.06-6.23) | -0.15(-0.34,0.03) |
| **Jordan** | 340.96(242.41-481.12) | 22.18(15.77-31.30) | 1277.59(908.88-1747.16) | 23.80(16.93-32.55) | -0.04(-0.26,0.18) |
| **Kazakhstan** | 871.64(535.28-1332.76) | 12.84(7.88-19.63) | 875.71(528.26-1375.90) | 12.57(7.58-19.74) | -0.53(-0.75,-0.30) |
| **Kenya** | 1216.90(807.43-1822.51) | 13.90(9.22-20.81) | 4213.14(2872.63-6321.04) | 19.46(13.27-29.19) | 1.36(1.18,1.54) |
| **Kiribati** | 11.15(7.64-15.80) | 36.53(25.04-51.76) | 25.21(15.11-39.47) | 50.71(30.39-79.37) | 0.77(0.58,0.95) |
| **Kuwait** | 247.94(176.55-336.09) | 29.34(20.89-39.77) | 400.52(281.23-529.55) | 18.87(13.25-24.95) | -1.50(-1.90,-1.10) |
| **Kyrgyzstan** | 276.87(177.74-422.44) | 15.35(9.85-23.42) | 384.41(247.69-562.80) | 14.12(9.10-20.68) | -0.82(-1.04,-0.61) |
| **Lao People's Democratic Republic** | 3354.37(2195.85-4604.41) | 217.11(142.13-298.02) | 6047.77(3917.27-9072.10) | 188.53(122.11-282.81) | -0.62(-0.70,-0.53) |
| **Latvia** | 59.88(40.75-87.87) | 6.28(4.27-9.21) | 40.34(27.21-61.51) | 7.49(5.05-11.42) | 0.17(-0.15,0.49) |
| **Lebanon** | 337.10(220.01-516.66) | 29.24(19.08-44.82) | 583.63(396.10-790.43) | 25.15(17.07-34.07) | -0.58(-0.87,-0.29) |
| **Lesotho** | 106.61(71.45-160.51) | 19.76(13.24-29.75) | 419.79(262.63-649.35) | 50.47(31.57-78.06) | 4.04(3.63,4.45) |
| **Liberia** | 749.90(442.31-1369.37) | 81.28(47.94-148.43) | 1947.11(1252.76-2794.89) | 86.72(55.80-124.48) | 0.87(0.36,1.38) |
| **Libya** | 455.68(299.65-660.40) | 27.12(17.83-39.30) | 1460.23(806.32-2203.90) | 48.67(26.87-73.45) | 2.60(2.27,2.93) |
| **Lithuania** | 100.62(69.08-149.99) | 7.22(4.96-10.77) | 65.46(44.97-96.75) | 8.13(5.59-12.02) | -0.11(-0.30,0.09) |
| **Luxembourg** | 13.22(8.07-20.69) | 8.96(5.47-14.02) | 17.76(9.44-28.65) | 8.05(4.28-12.99) | -0.65(-0.83,-0.48) |
| **Madagascar** | 900.82(611.09-1297.52) | 19.89(13.49-28.65) | 2271.58(1485.77-3379.05) | 19.40(12.69-28.85) | -0.07(-0.14,0.00) |
| **Malawi** | 958.07(639.86-1358.90) | 25.64(17.12-36.37) | 2489.24(1627.71-3612.35) | 30.42(19.89-44.14) | 0.49(0.24,0.74) |
| **Malaysia** | 6416.77(4846.83-8217.68) | 86.46(65.30-110.72) | 11536.66(8648.39-14970.09) | 82.98(62.21-107.68) | -0.41(-0.65,-0.18) |
| **Maldives** | 140.28(97.87-184.49) | 172.62(120.43-227.02) | 275.22(192.22-379.77) | 105.79(73.88-145.97) | -1.54(-1.84,-1.23) |
| **Mali** | 1885.98(1271.57-2767.47) | 63.17(42.59-92.69) | 5034.27(3310.92-7551.03) | 56.52(37.17-84.78) | -0.31(-0.46,-0.17) |
| **Malta** | 11.20(6.95-17.32) | 8.12(5.04-12.55) | 12.07(7.48-18.95) | 9.02(5.58-14.16) | 0.47(0.35,0.59) |
| **Marshall Islands** | 6.53(3.90-10.83) | 38.04(22.74-63.14) | 15.14(5.83-36.87) | 63.81(24.59-155.43) | 1.82(1.69,1.95) |
| **Mauritania** | 483.64(318.66-685.24) | 62.91(41.45-89.13) | 847.69(527.16-1304.31) | 49.66(30.88-76.41) | -0.97(-1.06,-0.87) |
| **Mauritius** | 766.62(599.74-955.66) | 154.21(120.64-192.24) | 1415.13(1113.37-1801.85) | 310.88(244.59-395.83) | 2.09(1.83,2.36) |
| **Mexico** | 8665.41(6587.81-11571.74) | 24.30(18.47-32.45) | 23052.46(16549.39-32212.50) | 44.75(32.13-62.53) | 2.38(2.17,2.59) |
| **Micronesia (Federated States of)** | 15.24(9.37-23.81) | 38.05(23.39-59.43) | 25.17(15.21-38.00) | 59.27(35.81-89.49) | 1.47(1.23,1.72) |
| **Monaco** | 0.60(0.39-0.93) | 6.57(4.26-10.15) | 0.67(0.40-1.05) | 7.23(4.33-11.31) | 0.11(-0.07,0.30) |
| **Mongolia** | 120.08(70.32-189.34) | 13.59(7.96-21.43) | 175.10(113.67-270.11) | 13.87(9.01-21.40) | -0.21(-0.34,-0.09) |
| **Montenegro** | 50.38(35.07-69.83) | 20.07(13.97-27.82) | 38.10(26.09-52.44) | 18.52(12.68-25.49) | -0.15(-0.40,0.09) |
| **Morocco** | 1651.21(1167.63-2412.83) | 15.88(11.23-23.21) | 2654.11(1845.89-3966.30) | 18.08(12.57-27.02) | 0.66(0.46,0.87) |
| **Mozambique** | 877.97(582.29-1338.41) | 18.51(12.28-28.22) | 3401.95(2099.23-5131.46) | 28.29(17.46-42.68) | 2.00(1.79,2.22) |
| **Myanmar** | 40718.86(26045.51-57841.59) | 237.19(151.72-336.93) | 38090.06(26093.08-53146.29) | 169.43(116.06-236.40) | -1.42(-1.60,-1.24) |
| **Namibia** | 118.95(77.70-205.34) | 21.27(13.89-36.72) | 287.10(170.60-480.15) | 27.47(16.32-45.94) | 0.48(0.07,0.88) |
| **Nauru** | 1.79(1.13-2.69) | 44.33(27.88-66.44) | 3.03(1.97-4.49) | 65.05(42.24-96.58) | 1.09(0.98,1.20) |
| **Nepal** | 2017.74(1421.87-2824.79) | 27.62(19.46-38.67) | 4297.87(2861.46-6093.99) | 32.06(21.34-45.45) | 0.19(-0.13,0.50) |
| **Netherlands** | 509.70(303.05-794.38) | 8.45(5.03-13.17) | 417.67(253.60-645.00) | 7.91(4.80-12.21) | -0.39(-0.50,-0.27) |
| **New Zealand** | 54.48(32.55-84.92) | 3.94(2.36-6.15) | 87.90(52.85-136.41) | 4.88(2.93-7.57) | 0.30(0.15,0.44) |
| **Nicaragua** | 402.09(292.03-549.08) | 27.24(19.78-37.20) | 1513.21(1003.33-2168.83) | 53.17(35.25-76.20) | 2.65(2.44,2.87) |
| **Niger** | 1311.67(845.50-2136.12) | 47.13(30.38-76.75) | 3447.06(2060.88-6122.28) | 38.66(23.11-68.66) | -0.85(-1.01,-0.69) |
| **Nigeria** | 12963.94(8511.15-18436.31) | 37.97(24.93-54.00) | 37240.33(22845.02-60747.91) | 41.41(25.40-67.54) | 0.34(0.15,0.54) |
| **Niue** | 0.26(0.16-0.38) | 31.74(20.14-46.86) | 0.35(0.20-0.57) | 61.53(35.18-99.91) | 1.43(1.20,1.66) |
| **North Macedonia** | 99.19(69.85-138.20) | 12.49(8.80-17.40) | 91.01(62.80-132.92) | 11.90(8.21-17.38) | 0.02(-0.12,0.15) |
| **Northern Mariana Islands** | 5.08(3.28-7.51) | 21.71(14.01-32.05) | 4.24(2.79-6.23) | 25.74(16.91-37.77) | 0.16(0.02,0.30) |
| **Norway** | 84.98(54.85-127.38) | 5.31(3.43-7.96) | 113.43(72.88-168.13) | 6.39(4.11-9.47) | 0.53(0.46,0.59) |
| **Oman** | 178.34(120.08-280.31) | 21.49(14.47-33.78) | 675.04(427.75-980.03) | 29.17(18.48-42.35) | 1.67(1.33,2.02) |
| **Pakistan** | 10700.54(7533.08-14990.69) | 26.22(18.46-36.74) | 41172.24(27959.72-58435.05) | 41.63(28.27-59.08) | 1.25(1.04,1.46) |
| **Palau** | 2.41(1.55-3.56) | 34.53(22.16-51.09) | 4.00(2.49-6.29) | 67.89(42.35-106.73) | 2.27(2.12,2.42) |
| **Palestine** | 217.05(144.68-329.85) | 28.28(18.85-42.98) | 606.93(433.76-816.18) | 27.79(19.86-37.38) | -0.29(-0.51,-0.08) |
| **Panama** | 150.51(106.49-210.11) | 14.88(10.53-20.77) | 394.20(278.52-550.63) | 23.88(16.88-33.36) | 1.68(1.29,2.07) |
| **Papua New Guinea** | 340.91(194.76-529.66) | 20.60(11.77-32.00) | 1084.74(732.14-1593.61) | 25.35(17.11-37.24) | 0.49(0.37,0.61) |
| **Paraguay** | 288.52(206.84-402.13) | 18.41(13.20-25.66) | 711.98(495.32-1013.19) | 23.27(16.19-33.12) | 0.73(0.63,0.83) |
| **Peru** | 2736.55(2021.35-3702.20) | 30.85(22.79-41.73) | 4128.46(2786.14-6023.64) | 27.78(18.75-40.53) | -0.49(-0.67,-0.30) |
| **Philippines** | 42826.61(33511.06-53788.75) | 165.23(129.29-207.53) | 97235.35(71629.17-124496.76) | 205.79(151.60-263.48) | 1.16(0.93,1.38) |
| **Poland** | 1966.47(1455.35-2637.63) | 13.61(10.08-18.26) | 814.27(572.76-1169.00) | 6.73(4.73-9.66) | -1.42(-1.77,-1.07) |
| **Portugal** | 360.02(250.11-525.53) | 9.51(6.61-13.88) | 217.21(140.53-321.51) | 7.36(4.76-10.89) | -0.79(-0.97,-0.61) |
| **Puerto Rico** | 483.88(362.01-656.60) | 34.21(25.59-46.42) | 398.35(318.60-491.03) | 38.51(30.80-47.48) | 0.61(0.34,0.89) |
| **Qatar** | 61.21(39.17-93.44) | 25.89(16.57-39.52) | 418.33(294.27-578.52) | 25.32(17.81-35.01) | -0.54(-0.88,-0.20) |
| **Republic of Korea** | 3194.12(2410.53-3949.93) | 15.18(11.45-18.77) | 1072.84(793.82-1455.39) | 6.70(4.96-9.10) | -2.23(-2.51,-1.94) |
| **Republic of Moldova** | 153.01(102.06-235.24) | 8.78(5.86-13.50) | 128.50(84.78-195.20) | 10.36(6.84-15.74) | 0.12(-0.07,0.31) |
| **Romania** | 716.87(495.89-1011.58) | 8.25(5.71-11.64) | 348.24(229.85-531.51) | 6.46(4.26-9.86) | -0.23(-0.53,0.07) |
| **Russian Federation** | 7060.38(5295.72-9568.83) | 12.14(9.10-16.45) | 3079.80(2226.72-4268.59) | 6.63(4.79-9.18) | -2.92(-3.23,-2.61) |
| **Rwanda** | 999.14(687.50-1459.54) | 36.46(25.09-53.26) | 1302.22(852.79-1940.27) | 22.96(15.04-34.21) | -2.38(-2.81,-1.95) |
| **Saint Kitts and Nevis** | 12.71(9.54-17.40) | 73.55(55.22-100.68) | 14.59(9.65-22.60) | 64.18(42.47-99.45) | -0.91(-1.56,-0.25) |
| **Saint Lucia** | 31.10(23.49-41.08) | 55.29(41.76-73.02) | 55.98(38.82-74.45) | 84.70(58.74-112.64) | 1.74(1.56,1.92) |
| **Saint Vincent and the Grenadines** | 22.33(16.77-29.54) | 48.62(36.52-64.31) | 31.40(22.15-42.30) | 75.98(53.60-102.37) | 1.77(1.50,2.04) |
| **Samoa** | 18.88(12.18-28.16) | 28.20(18.19-42.05) | 34.61(21.10-50.67) | 43.07(26.25-63.06) | 1.29(1.20,1.39) |
| **San Marino** | 0.53(0.31-0.86) | 5.61(3.31-9.13) | 0.52(0.28-0.86) | 5.76(3.14-9.55) | 0.15(-0.01,0.32) |
| **Sao Tome and Principe** | 29.63(17.23-43.68) | 69.01(40.14-101.75) | 86.36(49.72-137.71) | 95.02(54.71-151.52) | 0.81(0.38,1.25) |
| **Saudi Arabia** | 2378.47(1618.79-3502.08) | 35.79(24.36-52.70) | 15293.04(9031.49-23933.86) | 82.57(48.76-129.22) | 2.91(2.77,3.05) |
| **Senegal** | 1821.28(1205.48-2661.38) | 66.00(43.69-96.45) | 4233.38(2645.39-6831.33) | 65.66(41.03-105.95) | 0.21(0.02,0.40) |
| **Serbia** | 656.70(473.81-919.52) | 18.29(13.19-25.61) | 384.46(292.31-501.50) | 12.97(9.86-16.92) | -1.14(-1.27,-1.01) |
| **Seychelles** | 39.67(30.75-51.01) | 127.07(98.48-163.40) | 65.34(47.63-86.91) | 170.21(124.10-226.42) | 1.38(1.13,1.62) |
| **Sierra Leone** | 743.71(433.51-1186.63) | 46.56(27.14-74.28) | 1886.26(1203.65-3029.29) | 50.57(32.27-81.22) | 0.28(0.16,0.40) |
| **Singapore** | 96.15(69.12-132.42) | 6.37(4.58-8.77) | 104.38(69.45-157.49) | 5.43(3.61-8.19) | 0.19(-0.07,0.46) |
| **Slovakia** | 227.35(159.72-319.27) | 11.11(7.80-15.60) | 149.10(107.34-211.03) | 8.71(6.27-12.33) | -0.36(-0.69,-0.02) |
| **Slovenia** | 42.18(27.76-62.82) | 5.50(3.62-8.20) | 28.60(17.97-45.23) | 5.02(3.16-7.94) | -0.17(-0.24,-0.10) |
| **Solomon Islands** | 44.58(19.92-76.22) | 34.76(15.53-59.42) | 122.79(75.60-174.11) | 44.89(27.64-63.66) | 0.82(0.63,1.01) |
| **Somalia** | 869.17(543.34-1382.93) | 29.98(18.74-47.70) | 2499.02(1468.48-4153.17) | 30.14(17.71-50.09) | 0.27(0.15,0.38) |
| **South Africa** | 8485.10(5976.64-11285.65) | 53.92(37.98-71.72) | 15808.01(10710.97-21407.15) | 65.20(44.17-88.29) | -0.49(-1.29,0.31) |
| **South Sudan** | 475.50(312.35-701.49) | 20.60(13.53-30.39) | 1102.73(692.19-1677.42) | 30.64(19.23-46.61) | 1.24(0.93,1.55) |
| **Spain** | 1172.11(728.94-1757.88) | 7.90(4.92-11.85) | 810.30(493.20-1314.41) | 6.53(3.97-10.59) | -0.05(-0.29,0.18) |
| **Sri Lanka** | 9405.13(7254.26-12295.09) | 127.19(98.10-166.27) | 7730.43(5113.31-10955.94) | 95.86(63.40-135.85) | -1.38(-1.68,-1.08) |
| **Sudan** | 2570.82(1659.08-4084.71) | 33.74(21.77-53.60) | 7529.90(4448.24-10930.27) | 40.73(24.06-59.13) | 0.77(0.68,0.87) |
| **Suriname** | 93.67(64.82-127.29) | 57.52(39.80-78.17) | 191.27(128.22-271.67) | 89.11(59.73-126.56) | 1.44(1.22,1.67) |
| **Sweden** | 124.67(73.73-206.62) | 4.25(2.51-7.04) | 162.82(101.61-262.63) | 5.02(3.13-8.10) | 0.80(0.67,0.93) |
| **Switzerland** | 229.41(145.65-341.77) | 8.71(5.53-12.97) | 230.40(128.83-377.75) | 8.30(4.64-13.60) | -0.26(-0.38,-0.14) |
| **Syrian Arab Republic** | 2072.74(1416.71-2928.84) | 43.21(29.53-61.05) | 1786.56(1148.81-2944.30) | 35.13(22.59-57.89) | -0.48(-0.94,-0.02) |
| **Taiwan (Province of China)** | 3203.77(2476.11-4187.91) | 34.72(26.84-45.39) | 1745.62(1306.20-2356.40) | 23.14(17.31-31.23) | -0.94(-1.15,-0.73) |
| **Tajikistan** | 167.76(99.73-272.82) | 7.93(4.72-12.90) | 387.51(238.52-616.37) | 9.29(5.72-14.78) | 0.19(-0.02,0.39) |
| **Thailand** | 31227.51(22716.11-43880.07) | 120.42(87.60-169.21) | 29608.78(20367.90-42034.13) | 139.67(96.08-198.28) | -1.10(-1.89,-0.30) |
| **Timor-Leste** | 376.12(235.36-539.75) | 118.17(73.94-169.57) | 665.40(416.38-1024.75) | 116.46(72.88-179.36) | -0.07(-0.72,0.58) |
| **Togo** | 712.33(482.88-1007.06) | 51.96(35.22-73.46) | 2022.49(1294.45-3080.26) | 60.11(38.47-91.55) | 0.46(0.35,0.57) |
| **Tokelau** | 0.15(0.09-0.25) | 26.65(16.35-43.15) | 0.25(0.16-0.36) | 49.77(33.29-73.75) | 1.27(0.94,1.61) |
| **Tonga** | 5.45(3.58-7.82) | 14.78(9.71-21.18) | 8.22(5.62-11.65) | 21.14(14.44-29.95) | 1.14(1.04,1.23) |
| **Trinidad and Tobago** | 240.14(180.72-314.89) | 47.90(36.05-62.81) | 442.76(284.64-638.43) | 88.95(57.18-128.26) | 2.10(1.89,2.30) |
| **Tunisia** | 469.27(332.62-667.14) | 13.64(9.67-19.39) | 838.67(577.97-1201.70) | 19.27(13.28-27.61) | 1.08(0.97,1.19) |
| **Turkey** | 7246.67(5295.09-10218.75) | 30.30(22.14-42.72) | 6895.84(4931.00-9372.45) | 21.64(15.48-29.42) | -0.57(-0.85,-0.28) |
| **Turkmenistan** | 211.26(134.48-318.49) | 13.76(8.76-20.75) | 408.19(262.19-615.93) | 19.63(12.61-29.61) | 1.10(0.97,1.24) |
| **Tuvalu** | 1.25(0.84-1.86) | 34.62(23.14-51.45) | 2.19(1.43-3.34) | 44.06(28.69-67.18) | 0.71(0.65,0.78) |
| **Uganda** | 1024.39(682.49-1496.06) | 15.97(10.64-23.32) | 3856.26(2515.15-5848.65) | 22.42(14.62-34.01) | 0.56(0.29,0.84) |
| **Ukraine** | 741.31(414.50-1249.89) | 3.90(2.18-6.58) | 1235.89(853.54-1777.69) | 8.97(6.19-12.90) | 3.23(2.87,3.60) |
| **United Arab Emirates** | 225.67(156.22-324.48) | 23.61(16.34-33.94) | 1173.00(643.48-1712.34) | 29.20(16.02-42.62) | 0.76(0.55,0.96) |
| **United Kingdom** | 1706.93(1123.60-2519.82) | 8.17(5.38-12.06) | 1954.16(1280.97-2848.13) | 8.98(5.89-13.09) | -0.03(-0.21,0.16) |
| **United Republic of Tanzania** | 1341.01(957.57-1904.16) | 13.84(9.88-19.65) | 3412.78(2316.56-4791.95) | 14.63(9.93-20.54) | 0.13(0.07,0.19) |
| **United States of America** | 14576.15(10795.69-19343.71) | 14.27(10.57-18.93) | 30976.80(25353.91-36954.47) | 27.83(22.78-33.20) | 2.49(2.27,2.70) |
| **United States Virgin Islands** | 24.54(17.58-33.22) | 61.94(44.37-83.87) | 29.43(19.19-42.55) | 127.26(82.99-183.96) | 3.02(2.82,3.23) |
| **Uruguay** | 128.12(106.01-162.64) | 11.28(9.33-14.32) | 167.27(122.88-222.28) | 13.98(10.27-18.58) | 0.95(0.71,1.19) |
| **Uzbekistan** | 1065.11(657.67-1689.34) | 12.41(7.66-19.68) | 2497.73(1604.70-3802.06) | 18.18(11.68-27.68) | 0.98(0.83,1.13) |
| **Vanuatu** | 14.46(8.53-23.53) | 24.68(14.56-40.17) | 49.45(32.10-74.40) | 39.67(25.75-59.69) | 1.41(1.35,1.48) |
| **Venezuela (Bolivarian Republic of)** | 1425.06(1055.83-1910.39) | 17.79(13.18-23.85) | 2996.58(1993.16-4339.37) | 32.01(21.29-46.35) | 1.63(1.38,1.89) |
| **Viet Nam** | 32078.99(18383.27-44611.32) | 112.49(64.46-156.43) | 38889.03(24025.51-54679.74) | 101.30(62.58-142.43) | -0.28(-0.60,0.03) |
| **Yemen** | 763.99(447.45-1340.24) | 16.62(9.73-29.16) | 2258.93(1397.39-3780.32) | 16.42(10.16-27.47) | 0.09(-0.05,0.23) |
| **Zambia** | 891.36(611.61-1301.12) | 29.38(20.16-42.88) | 2951.50(1707.58-5058.18) | 36.47(21.10-62.50) | 0.50(0.38,0.62) |
| **Zimbabwe** | 853.48(564.60-1467.80) | 21.53(14.24-37.02) | 3104.70(1879.60-5179.47) | 48.99(29.66-81.73) | 3.39(2.70,4.08) |
| ***DALYs Disability-Adjusted Life Years,ASR Age-standardized rate, EAPC Estimated annual percentage change, CI Confidence interval, UI Uncertainty interval*** | | | | | |
